# Estimation of the average treatment effect with variable selection and measurement error simultaneously addressed for potential confounders

**DOI:** 10.1177/09622802221146308

**Published:** 2023-01-24

**Authors:** Grace Y. Yi, Li-Pang Chen

**Affiliations:** 1 Department of Statistical and Actuarial Sciences, 6221 University of Western Ontario, London, Canada; 2 Department of Computer Science, 6221 University of Western Ontario, London, Canada; 3 Department of Statistics, 34913 National Chengchi University, Taipei, Taiwan

**Keywords:** Causal inference, inverse probability weight, measurement error, propensity score, simulation–extrapolation, variable selection

## Abstract

In the framework of causal inference, the inverse probability weighting estimation method and its variants have been commonly employed to estimate the average treatment effect. Such methods, however, are challenged by the presence of irrelevant pre-treatment variables and measurement error. Ignoring these features and naively applying the usual inverse probability weighting estimation procedures may typically yield biased inference results. In this article, we develop an inference method for estimating the average treatment effect with those features taken into account. We establish theoretical properties for the resulting estimator and carry out numerical studies to assess the finite sample performance of the proposed estimator.

## 1 Introduction

Causal inference offers an important paradigm for answering a multitude of questions arising from a broad variety of areas such as healthcare, epidemiological studies, and social sciences. With observational studies, various estimation methods have been developed based on propensity scores, where the propensity score is defined as the conditional probability for an individual to receive a treatment, given pre-treatment confounders.^1^ In particular, the inverse probability weighting estimation methods are widely used due to their easy implementation and transparent interpretation (e.g. Rosenbaum and Rubin,^2^ Lunceford and Davidian,^3^ and Bang and Robins^4^). They adjust for the effects of measured confounders by re-weighting the data as if the weighted data were collected from randomized controlled trials.

The validity of those methods requires the treatment model to be correctly specified so that the propensity scores can be consistently estimated. To accommodate possible misspecification of the treatment model, Bang and Robins^4^ developed a doubly robust estimation method. This protection, nevertheless, does not come for free. It is at the price of specifying the outcome model correctly to facilitate the relationship between the outcome and confounders.

If correctly posing the outcome model is not possible, trying to postulate a feasible treatment model becomes necessary, which basically requires no omission of relevant pre-treatment confounders. Furthermore, to ensure the no-unmeasured-confounders assumption to be reasonable, one may tend to include as many variables as possible when building a treatment model. However, naively including all available variables in the treatment model would degrade inference results or even yield erroneous conclusions. In applications, some collected variables are unimportant in explaining the treatment variable, yet it is often unclear which variables should or should not be included in the treatment model, and we often rely on subject-matter knowledge to decide what variables are to be used for building a treatment model (e.g. Westreich et al.^5^). It is desirable to develop an analytical procedure for variable selection to build a suitable treatment model.

Utilizing variable selection techniques in causal inference has recently attracted interest. For example, Shortreed and Ertefaie^6^ proposed the outcome-adaptive lasso method to select covariates associated with outcomes. Ertefaie et al.^7^ developed a penalized objective function by employing both the outcome and treatment models to do a variable selection. Assuming Spike and slab priors for covariate coefficients, Koch et al.^8^ explored a Bayesian method to estimate causal effects with outcome and treatment models simultaneously employed. Ghosh et al.^9^ proposed the “multiply impute, then select” approach by employing the lasso method. Vansteelandt et al.^10^ utilized the focused information criterion to select important confounders.

While those methods are useful for formulating treatment models, their development hinges on an implicit but subtle condition that variables need to be precisely measured. This assumption, however, is commonly violated in applications. Measurement error arises inevitably and ubiquitously because of various reasons, such as the impossibility of measuring a long-term average quantity, inevitable recall bias in answering a questionnaire, unaffordability of precise measurements, the unwillingness of answering sensitive questions, and so on. Causal inference with measurement error has attracted extensive attention (e.g. Imai and Yamamoto^11^ and Edwards et al.^12^). For example, McCaffrey et al.^13^ and Shu and Yi^14,15^ proposed methods for adjusting measurement error effects on causal inference. To ameliorate measurement error effects, Kyle et al.^16^ explored the simulation–extrapolation (SIMEX) method for marginal structural models with time-varying covariates. These methods, however, do not consider variable selection to exclude unimportant pre-treatment variables.

In this article, we consider inverse probability weighting estimation in the presence of measurement error and unimportant pre-treatment variables. We focus on the circumstance where no prior knowledge is available to guide us to select relevant variables for building the treatment model and we rely on using variable selection techniques to do so. To provide valid estimation results, we propose a simulation-based estimation method for the average treatment effect. Variable selection for building the treatment model and accommodation of measurement error effects are simultaneously conducted in inferential procedures. Theoretical results for the proposed method are established rigorously. Our method has a broad scope of applications. It does not require modeling of the outcome process to specify the relationship of the outcome variable with confounders. The proposed method applies to any parametric models used to describe the treatment model. The implementation procedure is straightforward.

The rest of this article is organized as follows. Section 2 introduces the notation and inference framework. Section 3 reports the proposed method together with theoretical justifications. Simulation studies for the proposed method are included in Section 4 and an application of the proposed method is described in Section 5. Discussions and extensions are presented in the last section.

## 2 Notation and framework

For an individual, let *T* be the observed binary treatment variable, with T=1 if treated and T=0 if untreated. Let $Y_{\left(1\right)}$ denote the potential outcome that would have been observed had the subject been treated, and let $Y_{\left(0\right)}$ represent the potential outcome that would have been observed had the subject been untreated. We are interested in estimating the average treatment effect (ATE), $\tau_{0}≜E(Y_{\left(1\right)})-E(Y_{\left(0\right)})$ . While *T* is generically termed as a treatment indicator here, its practical meaning varies in applications. For example, *T* can be used to represent the exposure to a certain condition, as the case in the example presented in Section 5.

Let *Y* represent the observed outcome for an individual, which is assumed to be linked with potential outcomes via $Y=TY_{\left(1\right)}+(1-T)Y_{\left(0\right)}$ . This assumption, called *consistency*, basically says that the potential outcome under the observed treatment status equals the observed outcome.

In a randomized trial, the treatment indicator *T* is determined randomly so the potential outcomes $\left\{Y_{\left(1\right)},Y_{\left(0\right)}\right\}$ and *T* are independent. Consequently, the sample average of the response measurements for the treated and untreated groups can be directly used to estimate $\tau_{0}$ by taking their difference. However, in a non-randomized trial or an observational study, the relationship $E(Y_{\left(j\right)})=E\{Y_{\left(j\right)}|T=j\}$ does not hold anymore for j=0,1 due to the presence of confounders that are not controlled for the treated and untreated groups.

Let *W* be the vector of pre-treatment covariates or confounders. We assume that *W* contains all confounders associated with both potential outcomes and treatment and that only some components in *W* are predictive for *T*. That is, *W* can be written as $W=(W_{I}^{T},W_{II}^{T}{)}^{T}$ so that $\left\{Y_{\left(1\right)},Y_{\left(0\right)}\right\}$ and *T* are conditionally independent, given *W*; and $P(T=1|W)=P(T=1|W_{I})$ . In other words, $W_{I}$ includes important variables to predict *T*, whereas $W_{II}$ contains unimportant variables for predicting *T*.

The introduction of *W* allows us to express $E(Y_{\left(j\right)})$ using the observed outcome *Y* and the treatment indicator *T*. Specifically, let $\pi ≜P(T=1|W_{I})$ denote the *propensity score* for an individual, then with the *consistency* assumption, we obtain that

$$\begin{array}{rl} E\left(\frac{TY}{\pi }\right) & =E\left[\frac{T\{TY_{\left(1\right)}+(1-T)Y_{\left(0\right)}\}}{\pi }\right]\\ & =E\left\{\frac{TY_{\left(1\right)}}{\pi }\right\}\\ & =E\left[\frac{E\{TY_{\left(1\right)}|W\}}{\pi }\right]\\ & =E\left\{\frac{E(T=1|W)E\{Y_{\left(1\right)}|W\}\;}{\pi }\right\}\\ & =E\left\{\frac{E(T=1|W_{I})E\{Y_{\left(1\right)}|W\}\;}{\pi }\right\}\\ & =E\{E(Y_{\left(1\right)}|W)\}\\ & =E\{Y_{\left(1\right)}\} \end{array}$$

where the second step comes from that $T^{2}=T$ and T(1−T)=0 , the fourth step is due to the assumption of no unmeasured confounders, and the fifth step results from the nature of $W_{I}$ .

Similarly, we obtain that $E\{(1-T)Y/\left(1-\pi \right)\}=E\{Y_{\left(0\right)}\}$ , and thus, $E\{(TY/\pi )-[(1-T)Y/\left(1-\pi \right)]\}=\tau_{0}$ . These identities provide us a basis for constructing a consistent estimator of $\tau_{0}$ using the observed data, as described below. Here the *positivity* assumption 0<π<1 is implicitly made to ensure the denominators are meaningful.

### 2.1 Consistent estimator

To estimate $\tau_{0}$ , suppose we have a sample $\left\{(T_{i},Y_{i},W_{i})\;:\;i=1,\ldots ,n\right\}$ of size *n*, where $T_{i},Y_{i}$ , and $W_{i}=(W_{Ii}^{T},W_{IIi}^{T}{)}^{T}$ represent the corresponding variables for subject *i* in the sample for i=1,…,n . To use the confounder information together with the sample response measurements obtained from observational studies, Rosenbaum and Rubin^1^ initiated the propensity score method to balance the distribution of the covariates for the treated and untreated groups. For i=1,…,n , let

$$\pi_{i}=P(T_{i}=1|W_{Ii})$$

denote the conditional probability for subject *i* to receive the treatment, given the predictive covariates $W_{Ii}$ ; this probability is called the *propensity score* of subject *i*.

If $\pi_{i}$ can be consistently estimated, say, by ${\hat{\pi }}_{i}$ , for subject i=1,…,n , then a consistent estimator of $\tau_{0}$ can be constructed by

(1) $$\hat{\tau }=\frac{1}{n}\sum_{i=1}^{n}\frac{T_{i}Y_{i}}{{\hat{\pi }}_{i}}-\frac{1}{n}\sum_{i=1}^{n}\frac{(1-T_{i})Y_{i}}{1-{\hat{\pi }}_{i}}.$$

To mitigate unstable numerical results caused by extreme ${\hat{\pi }}_{i}$ close to 0 or 1, Lunceford and Davidian^3^ proposed a stable version of (1), which is also consistent:

(2) $$\hat{\tau }={\left(\sum_{i=1}^{n}\frac{T_{i}}{{\hat{\pi }}_{i}}\right)}^{-1}\sum_{i=1}^{n}\frac{T_{i}Y_{i}}{{\hat{\pi }}_{i}}-{\left(\sum_{i=1}^{n}\frac{1-T_{i}}{1-{\hat{\pi }}_{i}}\right)}^{-1}\sum_{i=1}^{n}\frac{(1-T_{i})Y_{i}}{1-{\hat{\pi }}_{i}}.$$

We use (2) for the following development.

### 2.2 Variable selection and measurement error

The consistency of the estimator (2) hinges on the consistent estimation of the propensity score $\pi_{i}$ for subject i=1,…,n . While the propensity score $\pi_{i}$ is assumed to be determined by $W_{Ii}$ but not $W_{IIi}$ , in building the treatment model for $\pi_{i}$ , it is usually unclear what components of $W_{i}$ should be included in $W_{Ii}$ , though subject matter knowledge is often utilized to help. Here we consider the circumstance where no prior knowledge is available to guide us to form $W_{Ii}$ and we rely on using variable selection techniques to decide what variables in $W_{i}$ are to be selected to form $W_{Ii}$ . Without loss of generality, components in $W_{i}$ are assumed to be standardized to have mean 0 and variance 1.

Suppose that we start with postulating the propensity score $\pi_{i}$ using a parametric model, denoted $g(W_{i};\gamma )$ , with all the components $W_{Ii}$ and $W_{IIi}$ in $W_{i}$ included:

(3) $$\pi_{i}=g(W_{Ii},W_{IIi};\gamma ),$$

where g(⋅) is a link function, such as the logit, probit, or complementary log–log function; and $\gamma =(\gamma_{0},\gamma_{I}^{*T},\gamma_{II}^{T}{)}^{T}$ is the vector of regression parameters, with $\gamma_{0}$ representing the intercept and $\gamma_{I}^{*}$ and $\gamma_{II}$ standing for the parameters, respectively, corresponding to $W_{Ii}$ and $W_{IIi}$ . The importance of $W_{Ii}$ and irrelevance of $W_{IIi}$ with respect to $T_{i}$ is then reflected by that $\gamma_{I}^{*}\neq 0$ and $\gamma_{II}=0$ . Here and elsewhere, we loosely use 0 to denote a zero vector whose dimension is inferred from the context. In other words, to identify salient variables in $W_{i}$ , one may apply variable selection techniques to model (3) and then examine estimates for the components in γ to determine what components in $W_{i}$ need to be selected to form $W_{Ii}$ . For ease of exposition, we write $\gamma_{I}=(\gamma_{0},\gamma_{I}^{*T}{)}^{T}$ and $\gamma =(\gamma_{I}^{T},\gamma_{II}^{T}{)}^{T}$ ; let *p* denote the dimension of $(\gamma_{I}^{*T},\gamma_{II}^{T}{)}^{T}$ ; and we also write $\gamma =(\gamma_{0},\gamma_{I}\ldots \;\gamma_{p}{)}^{T}$ .

The estimation of γ may proceed as follows. Let $S_{i}(\gamma ;W_{i})$ denote the likelihood score function, or more generally, an unbiased estimating function, derived from model (3) to reflect the contribution from subject *i* in the sample. If the true value of $W_{i}$ is available, one may solve

(4) $$\sum_{i=1}^{n}S_{i}(\gamma ;W_{i})=0$$

for γ to obtain a consistent estimator under regularity conditions.

In applications, some variables in $W_{i}$ are error-prone and their accurate measurements are not available for all subjects in the study. To characterize this feature, we re-write $W_{Ii}=(Z_{Ii}^{T},X_{Ii}^{T}{)}^{T}$ and $W_{IIi}=(Z_{IIi}^{T},X_{IIi}^{T}{)}^{T}$ so that $Z_{i}≜(Z_{Ii}^{T},Z_{IIi}^{T}{)}^{T}$ represents the subvector of error-free covariates in $W_{i}$ and $X_{i}≜(X_{Ii}^{T},X_{IIi}^{T}{)}^{T}$ is the subvector of error-prone covariates in $W_{i}$ . Furthermore, let $X_{i}^{*}=(X_{Ii}^{*T},X_{IIi}^{*T}{)}^{T}$ denote the observed version of $X_{i}$ , where $X_{Ii}^{*}$ and $X_{IIi}^{*}$ are the observed measurements of $X_{Ii}$ and $X_{IIi}$ , respectively.

Suppose that $X_{i}^{*}$ and $X_{i}$ are linked by the classical additive error model

(5) $$X_{i}^{*}=X_{i}+e_{i},$$

where the error term $e_{i}$ is independent of $\left\{T_{i},X_{i},Z_{i},Y_{(0)i},Y_{(1)i}\right\}$ and follows $N(0,\Sigma_{e})$ with covariance matrix $\Sigma_{e}$ . Here $Y_{(1)i}$ (or $Y_{(0)i}$ ) represents the potential outcome that would have been observed had subject *i* been treated (or untreated). Model (5) features situations where the observed value fluctuates around the true value with an error term; this model is most commonly used in the literature (e.g. Carroll et al.^17^ and Yi^18^).

While model (5) is useful to describe measurement error problems in applications, its introduction brings in an issue of parameter non-identifiability (e.g. Yi^18^ and Yi et al.^19^). To circumvent this issue and highlight the main ideas, we assume that $\Sigma_{e}$ is known for now and defer the discussions on handling unknown $\Sigma_{e}$ to Section 6.

## 3 Simulation-based method

In this section, we develop a method of estimating the average treatment effect $\tau_{0}$ by incorporating the features of variable selection and measurement error. We also establish the asymptotic properties of the resulting estimator.

### 3.1 Implementation procedure

Here we describe a simulation-based method that applies to any parametric form of the treatment model (3). The method roots in the SIMEX algorithm developed for the non-causal inference framework by Cook and Stefanski,^20^ but it extends the scope of the usual SIMEX algorithm by including extra steps for variable selection and inverse probability treatment weighting estimation. The estimation procedure consists of five steps. The first three steps come from the SIMEX method which aims to correct for measurement error effects on the estimation of the parameters associated with the treatment model (3), the fourth step performs the selection of active variables for building a suitable treatment model, and the last step outputs a valid estimator of $\tau_{0}$ . The five implementation steps are described as follows, and the programming code is available at GitHub: https://github.com/lchen723/Causal-inference-R-code.git.

**Step 1. Simulation:**

Let *K* be a given positive integer (say, K=500 ) and let $C=\{\psi_{1},\psi_{2},\ldots ,\psi_{M}\}$ be a sequence of *M* (say, M=10 ) non-negative numbers taken from $\left[0,\psi_{M}\right]$ with a given $\psi_{M}$ (say, $\psi_{M}=1$ ), where $\psi_{1}=0$ . For k=1,…,K , independently generate random variates $e_{ik}$ from $N(0,\Sigma_{e})$ and define

$$X_{i}^{*}(k,\psi )=X_{i}^{*}+\sqrt{\psi }e_{ik}\;\mathrm{for}\;\psi \in C.$$

**Step 2. Estimation of treatment model parameters**:

In (4), we replace $X_{i}$ with $X_{i}^{*}(k,\psi )$ and solve

$$\sum_{i=1}^{n}S_{i}(\gamma ;X_{i}^{*}(k,\psi ))=0$$

for γ to obtain an estimator, denoted $\hat{\gamma }(k,\psi )$ , where the dependence of $S_{i}(\gamma ;X_{i}^{*}(k,\psi ))$ on $Z_{i}$ is suppressed in the notation. Next, calculate

$$\hat{\gamma }(\psi )=K^{-1}\sum_{k=1}^{K}\hat{\gamma }(k,\psi ).$$

**Step 3. Extrapolation**:

For j=0,1,…,p , let ${\hat{\gamma }}_{j}(\psi )$ denote the *j*th element of $\hat{\gamma }(\psi )$ ; fit a regression model to $\left\{(\psi ,{\hat{\gamma }}_{j}(\psi ))\;:\;\psi \in C\right\}$ and extrapolate it to ψ=−1 ; and let ${\tilde{\gamma }}_{j}$ denote the resulting extrapolated estimator of $\gamma_{j}$ , the *j*th element of γ . Write $\tilde{\gamma }=({\tilde{\gamma }}_{0},{\tilde{\gamma }}_{1},\ldots ,{\tilde{\gamma }}_{p}{)}^{T}$ . 

**Step 4. Variable selection**:

Define a quadratic loss function:

(6) $$\ell (\gamma )=\frac{1}{2}(\gamma -\tilde{\gamma }{)}^{T}V_{n}(\gamma -\tilde{\gamma }),$$

where $V_{n}$ is a user-specified positive definite weight matrix. Different specifications of $V_{n}$ may result in estimators having different efficiency; taking $V_{n}$ to be the covariance matrix of $\tilde{\gamma }$ yields an efficient result, and setting $V_{n}$ as an identity matrix gives an easy implementation.

Consider the penalized quadratic loss function:

(7) $$\ell_{P}(\gamma )=\ell (\gamma )-n\sum_{\;j=1}^{p}p_{\lambda }(|\gamma_{\;j}|),$$

where $p_{\lambda }(\cdot )$ is a penalty function with a tuning parameter λ . Here we consider a weighted $L_{1}$ -penalty^21^:

$$p_{\lambda }(|\gamma_{\;j}|)=p_{\lambda }^{\prime }(|{\tilde{\gamma }}_{\;j}|)|\gamma_{\;j}|,$$

where $p_{\lambda }^{\prime }(u)$ is the first order derivative of $p_{\lambda }(u)$ , and $p_{\lambda }(u)$ can be set as a commonly used penalty function such as the least absolute shrinkage and selection operator (LASSO) penalty^22^ or the smoothly clipped absolute deviation (SCAD) penalty^23^ that are commonly used in applications. With the LASSO penalty, we set

$$p_{\lambda }(|\gamma_{\;j}|)=\lambda |\gamma_{\;j}|,$$

and for the SCAD penalty, we take

$$p_{\lambda }^{\prime }(x)=\lambda \{I(x\leq \lambda )+\frac{(a\lambda -x{)}_{+}}{(a-1)\lambda }\cdot I(x>\lambda )\},$$

where I(⋅) is the indicator function, $x_{+}=\max\{x,0\}$ , and a=3.7 .

To achieve a satisfactory performance of the selection procedure, we consider a grid Λ of possible values for the tuning parameter λ , and for λ∈Λ , let $\hat{\gamma }(\lambda )={\mathrm{argmin}}_{\gamma }\ell_{P}(\gamma )$ and let $df_{\lambda }$ denote the number of non-zero elements of $\hat{\gamma }(\lambda )$ . We define

$$BIC(\lambda )=-2\ell (\hat{\gamma }(\lambda ))+2(\log\;n)df_{\lambda }.$$

Then the optimal tuning parameter $\lambda^{*}$ is chosen as the minimizer of BIC(λ) :

$$\lambda^{*}={\mathrm{argmin}}_{\lambda \in \Lambda }BIC(\lambda )$$

and the resulting estimator $\hat{\gamma }(\lambda^{*})$ , denoted $\hat{\gamma }$ , is taken as the estimator of γ .

**Step 5. Estimation of ATE**:

Write $\hat{\gamma }=({\hat{\gamma }}_{I}^{T},{\hat{\gamma }}_{II}^{T}{)}^{T}$ with ${\hat{\gamma }}_{I}=({\hat{\gamma }}_{0},{\hat{\gamma }}_{xI}^{T},{\hat{\gamma }}_{zI}^{T}{)}^{T}$ and ${\hat{\gamma }}_{II}=({\hat{\gamma }}_{xII}^{T},{\hat{\gamma }}_{zII}^{T}{)}^{T}$ , respectively, corresponding to the non-zero and zero components in $\hat{\gamma }$ . Importance of covariates $\left\{X_{Ii},Z_{Ii}\right\}$ and unimportance of covariates $\left\{X_{IIi},Z_{IIi}\right\}$ are thereby suggested by the estimates of the corresponding coefficients. With unimportant variables $X_{IIi}$ and $Z_{IIi}$ excluded from the initial model (3), the final treatment model is taken as

(8) $$\pi_{i}=g(X_{Ii},Z_{Ii};\gamma_{I}),$$

where $\gamma_{I}$ includes the intercept and the model parameters associated with important covariates $\left\{X_{Ii},Z_{Ii}\right\}$ .

Let $X_{Ii}^{*}(k,\psi )$ denote the subvector of $X_{i}^{*}(k,\psi )$ corresponding to $X_{Ii}^{*}$ , generated from Step 1. Using the selected treatment model (8) with $X_{Ii}$ replaced by $X_{Ii}^{*}(k,\psi )$ , we calculate the fitted value ${\hat{\pi }}_{i}(k,\psi )≜g(X_{Ii}^{*}(k,\psi ),Z_{Ii};{\hat{\gamma }}_{I})$ . Then we obtain an estimate, say, $\hat{\tau }(k,\psi )$ , of $\tau_{0}$ using (2) with ${\hat{\pi }}_{i}$ replaced by ${\hat{\pi }}_{i}(k,\psi )$ , and calculate

$$\hat{\tau }(\psi )=K^{-1}\sum_{k=1}^{K}\hat{\tau }(k,\psi ).$$

Finally, we fit a regression model to $\left\{(\psi ,\hat{\tau }(\psi ))\;:\;\psi \in C\right\}$ and extrapolate it to ψ=−1 . The resulting value, denoted as $\hat{\tau }$ , is taken as an estimate of $\tau_{0}$ .

We conclude this section with a few remarks. The basic idea of the implementation roots in using the available estimation method (2), which is developed under the ideal situation where all the pre-treatment covariates are relevant and measured without error. We start with an initial treatment model (3) by using all pre-treatment variables in $W_{i}$ to express propensity scores.

The first three steps are directed to addressing the measurement error effects in the estimation of the propensity scores; and the last step estimates the ATE $\tau_{0}$ with the measurement error effects accounted for by employing the SIMEX algorithm. The idea of the SIMEX is to first establish the trend of measurement error-induced biases as a function of the variance of measurement error by artificially creating a sequence of surrogate measurements, and then extrapolate this trend back to the case without measurement error. Specifically, in Step 1, we artificially create a sequence of error-contaminated surrogate measurements by introducing different degrees of measurement error, and then we apply those surrogate measurements in Step 2 to obtain biased estimates by running an estimation method developed for error-free settings. Step 3 traces the patten of biased estimates against varying magnitudes of measurement error and then does extrapolation. To analytically demonstrate this rationale, one may consider the simple linear regression model with an additive measurement error in the covariate; an intuitive illustration of the idea can be found in Yi.^18^ ${}^{\left(p.\;63-64\right)}$ In implementing Steps 3 and 5, it is ideal to use the true extrapolation function as is required in establishing the theoretical results in the next subsection. However, the exact function form is typically unknown in applications, and one has to invoke a specified regression model to approximate it. As a result, this approximation makes the resulting SIMEX estimators not exactly but only approximately consistent. Further, this suggests that the performance of the SIMEX estimators can be sensitive to the choice of a working extrapolation function form. In our simulation studies to be reported in Section 4, we compare how different specifications of the extrapolation function in Steps 3 and 5 may affect the performance of the estimation.

Step 4 requires the optimal value for the tuning parameter λ . While different criteria such as generalized cross validation (GCV) and Akaike information criterion (AIC) are commonly used in applications, Wang et al.^24^ and Zhang et al.^25^ showed that the optimal tuning parameter derived from the AIC and GCV criteria has a nonignorable overfitting effect and that the tuning parameter derived from the Bayesian information criterion (BIC) with the SCAD can identify the true model consistently under linear or partial linear regression models. Here we consider a criterion based on the BIC following Yi et al.^26^ and Chen and Yi.^27^

### 3.2 Theoretical results

Now we justify the validity of the algorithm described in Section 3.1 by establishing asymptotic properties for the associated estimators. Consistent with authors such as Fan and Li,^23^ Yi et al.,^26^ and Carroll et al.,^28^ the following regularity conditions are considered:

(A).  As n→∞ , n−1Vnp⟶V , where *V* is positive definite.
(B).  As n→∞ , n(γ~−γ)d⟶N(0,Σ) , where Σ is positive definite.
(C).  To express the dependence on the sample size, we write the tuning parameter as $\lambda_{n}$ . Define
$$a_{n}=\max\left\{\;p_{\lambda_{n}}^{\prime }(|\gamma_{j}|)\;:\gamma_{j}\neq 0\;\mathrm{with}\;j=1,\ldots ,p\right\}\;\mathrm{and}\;b_{n}=\max\left\{\;p_{\lambda_{n}}^{\prime \prime }(|\gamma_{j}|):\gamma_{j}\neq 0\;\mathrm{with}\;j=1,\ldots ,p\right\}.$$
  Assume that as n→∞ ,
$$a_{n}\to 0\;\text{and}\;b_{n}\to 0\;\;\mathrm{and}\;{\text{liminf}}_{n\to \infty }{\text{liminf}}_{u\to 0^{+}}\sqrt{n}p_{\lambda_{n}}^{\prime }(u)=\infty .$$
(D).  The extrapolation function in Step 5 is correctly specified.

Repeating the proof for Theorem 1 of Fan and Li,^23^ we can show that with regularity conditions, there exists an estimator $\hat{\gamma }$ such that

$$\|\hat{\gamma }-\gamma \|=O_{p}(n^{-1/2}+a_{n}).$$

This suggests that $\hat{\gamma }$ is a $\sqrt{n}$ -consistent estimator of γ if $a_{n}=O_{p}(n^{-1/2})$ .

Next, we discuss the asymptotic properties of $\hat{\gamma }$ . Split *V* into a 2×2 block matrix:

$$V=\left(\begin{array}{cc} V_{I,I} & V_{I,II}\\ V_{II,I} & V_{II,II} \end{array}\right),$$

where $V_{u,v}$ is the submatrix of dimension $p_{u}\times p_{v}$ for **u,v=I,II ,** $p_{I}$ represents the dimension of $\gamma_{I}$ , and $p_{II}$ stands for the dimension of $\gamma_{II}$ . Write $V_{I}=[V_{I,I}\;V_{I,II}]$ . Using the arguments of Yi et al.,^26^ we can prove the following results.

Theorem 3.1.
*Assume that regularity conditions (A) to (C) hold. Then the following results hold:*

(a).  As n→∞ ,
$$\sqrt{n}({\hat{\gamma }}_{I}-\gamma_{I})\overset{d}{\to }N(0,V_{I,I}^{-1}V_{I}\Sigma V_{I}^{T}V_{I,I}^{-1}).$$
(b). 
${\hat{\gamma }}_{zII}=0$ and ${\hat{\gamma }}_{xII}=0$ .
(c).  When *V* is equal to $\Sigma^{-1}$ , the covariance matrix of ${\hat{\gamma }}_{I}$ is no greater than the covariance variance of ${\tilde{\gamma }}_{I}$ in the Loewner order, where ${\tilde{\gamma }}_{I}$ is the subvector of the SIMEX estimator $\tilde{\gamma }$ corresponding to $\gamma_{I}$ .

Theorem 3.1(a) establishes the asymptotic distribution for the estimators for the effects corresponding to important pre-treatment variables in model (3), or equivalently, for the estimators of the parameters for the selected treatment model (8). Theorem 3.1(b) ensures the oracle property in the sense of Fan and Li^23^ for the variable selection procedure for building the final treatment model (8). The results in Theorem 3.1(a) and (b) are established in the same lines as Zou and Li^21^ and Fan and Li,^23^ which basically require regularity condition (C). This condition is satisfied by the SCAD penalty but not the LASSO penalty. Condition (B) is needed in showing Theorem 3.1(a), and its validity is ensured by the result of Carroll et al.,^28^ who assumed the knownness of the extrapolation function in Step 3.

Theorem 3.1(c) suggests that the estimator obtained from conducting an extra step of variable selection (i.e. obtained from Steps 1–4) is more efficient than the usual SIMEX estimator (i.e. obtained from Steps 1–3). This shows the necessity and importance of excluding inactive pre-treatment variables when building the treatment model. Furthermore, we show the following asymptotic distribution of the estimator $\hat{\tau }$ established in Step 5 of Section 3.1, and its proof is deferred to the Appendix.

Theorem 3.2.
*Assume that regularity conditions (A) to (D) hold. Then the estimator* $\hat{\tau }$ *obtained in Section 3.1 has the asymptotic distribution*

$$\sqrt{n}(\;\hat{\tau }-\tau_{0})\overset{d}{\to }N(0,v(\tau_{0}))\;\mathrm{as}\;n\to \infty ,$$

where $v(\tau_{0})$ is defined in the Appendix.

## 4 Simulation studies

In this section, we conduct simulation studies to assess the finite sample performance of the proposed method. As described in Section 3, we first select the variables and estimate the associated parameters for the treatment model, and then estimate the average treatment effect $\tau_{0}$ . For each parameter configuration, we repeat the simulation 500 times, where the sample size is set as n=400 .

### 4.1 Simulation designs

We consider one of the following two outcome models, which show different types of dependence of *Y* on the covariates {X,Z} and the treatment indicator variable *T*:

$$\begin{array}{rl} \mathrm{Model}\;\;1\;:\; & \;Y=T+\beta_{x}^{T}X+\beta_{z}^{T}Z+\epsilon \\ \mathrm{Model}\;\;2\;:\; & \;\mathrm{logit}\;P(Y=1|T,X,Z)=T+\beta_{x}^{T}X+\beta_{z}^{T}Z \end{array}$$

where ϵ is independent of {X,Z,T} ; ϵ∼N(0,1) ; $\beta_{x}$ and $\beta_{z}$ are the model parameters of dimension $p_{x}$ and $p_{z}$ , respectively. Here we take $\beta_{x}=(1_{d_{x}}^{T}\;0_{\;p_{x}-d_{x}}^{T}{)}^{T}$ and $\beta_{z}=(1_{d_{z}}^{T}\;0_{\;p_{z}-d_{z}}^{T}{)}^{T}$ with $d_{x}≜\lceil \frac{\;p_{x}}{2}\rceil$ and $d_{z}≜\lceil \frac{\;p_{z}}{2}\rceil$ , where ⌈a⌉ represents the ceiling function of *a*, that is, it is the least integer that is ≥a for a real number *a*; and $1_{r}$ and $0_{r}$ represent the r×1 unit and zero vectors, respectively, for a positive integer *r*. We set $p_{x}$ and $p_{z}$ both to be 15, yielding that Models 1 and 2 include 16 important covariates of which 8 are error-prone, and 14 unimportant covariates of which 7 are error-prone.

The treatment indicator $T_{i}$ is independently generated from the treatment model (3) for i=1,…,n . We consider three useful model forms for (3), respectively, given by

- *logistic regression model:*
(9) $$\pi_{i}=\frac{\exp\;\left(\gamma_{0}+X_{i}^{T}\gamma_{x}+Z_{i}^{T}\gamma_{z}\right)}{1+\exp\;\left(\gamma_{0}+X_{i}^{T}\gamma_{x}+Z_{i}^{T}\gamma_{z}\right)}$$
- *probit regression model:*
(10) $$\pi_{i}=\Phi (\gamma_{0}+X_{i}^{T}\gamma_{x}+Z_{i}^{T}\gamma_{z})$$
- *complementary log–log regression model:*
(11) $$\pi_{i}=1-\exp\;\{-\exp\;(\gamma_{0}+X_{i}^{T}\gamma_{x}+Z_{i}^{T}\gamma_{z})\}$$

where $\gamma =(\gamma_{0},\gamma_{x}^{T},\gamma_{z}^{T}{)}^{T}$ is the vector of parameters, and Φ(⋅) is the cumulative standard normal distribution function.

For all those treatment models, consider the case with $\gamma_{x}=\gamma_{z}=(1_{d_{x}/2}^{T},\;-1_{d_{x}/2}^{T},\;0_{\;p_{x}-d_{x}}^{T}{)}^{T}$ and $\gamma_{0}=1$ . Thus, the number of important error-prone and important error-free variables is both 8, and the number of unimportant error-prone and unimportant error-free variables is both 7.

To generate measurements of $Y_{i}$ and $T_{i}$ , respectively, from an outcome and treatment model independently for i=1,…,n , we need first to simulate measurements for the covariates $\left\{X_{i},Z_{i}\right\}$ as well as for the treatment indicator variable $T_{i}$ . For i=1,…,n , we generate covariate measurements independently from the normal distribution: $\left(X_{i}^{T},Z_{i}^{T}{)}^{T}∼N(0_{\;p},\Sigma_{w}\right)$ , where the matrix $\Sigma_{w}$ is written as

$$\left(\begin{array}{cc} \Sigma_{xx} & \Sigma_{xz}\\ \Sigma_{zx} & \Sigma_{zz} \end{array}\right)$$

with $\Sigma_{xz}=\Sigma_{zx}$ and $\Sigma_{xx}$ and $\Sigma_{zz}$ , respectively, representing the covariance matrices for $X_{i}$ and $Z_{i}$ . Let $\sigma_{xzjk}$ , $\sigma_{xjk}$ , and $\sigma_{zjk}$ denote element (j,k) of $\Sigma_{xz}$ , $\Sigma_{xx}$ , and $\Sigma_{zz}$ , respectively. We consider the case with $\sigma_{xzjk}=0.4^{\left(2+|j-k|\right)}$ , $\sigma_{xjk}=\sigma_{x}^{2}\rho_{x}^{\left|j-k\right|}$ , and $\sigma_{zjk}=\sigma_{z}^{2}\rho_{z}^{\left|j-k\right|}$ by setting $\sigma_{x}^{2}=\sigma_{z}^{2}=1.0$ and $\rho_{x}=\rho_{z}=0.5$ .

To generate surrogate measurements $X_{i}^{*}$ for $X_{i}$ , we consider the classical additive error model (5), where $\Sigma_{e}$ is set as a diagonal matrix with a common element $\sigma_{e}^{2}$ . We set $\sigma_{e}^{2}$ to be 0.15, 0.50, or 0.75, yielding the signal-to-noise ratio $\mathrm{var}(X_{j})/\mathrm{var}(X_{j}^{*})$ to be 44, 4, and 1.8, respectively.

Finally, we examine the value of the average treatment effect, $\tau_{0}$ , under each of the outcome models. Model 1 shows a scenario with continuous outcomes and yields the true value $\tau_{0}$ to be

$$\begin{array}{rl} \tau_{0} & =E(Y_{\left(1\right)})-E(Y_{\left(0\right)})=E\{E(Y_{\left(1\right)}|T,X,Z)\}-E\{E(Y_{\left(0\right)}|T,X,Z)\}\\ & =E(1+\beta_{x}^{T}X+\beta_{z}^{T}Z+\epsilon )-E(0+\beta_{x}^{T}X+\beta_{z}^{T}Z+\epsilon )=1 \end{array}$$

On the contrary, Model 2 forms a logistic regression model for binary outcomes, yielding the true value $\tau_{0}$ to be

(12) $$\begin{array}{rl} \tau_{0} & =P(Y_{\left(1\right)}=1)-P(Y_{\left(0\right)}=1)\\ & =\int_{R^{\;p_{x}}}\int_{R^{\;p_{z}}}P(Y=1|T=1,X=x,Z=z)f(x,z)dzdx\\ & \;-\int_{R^{\;p_{x}}}\int_{R^{\;p_{z}}}P(Y=1|T=0,X=x,Z=z)f(x,z)dzdx \end{array}$$

where f(x,z) is the density function of {X,Z} , which is specified as a multivariate normal distribution earlier, and P(Y=1|T=1,X=x,Z=z) is determined by Model 2. As (12) does not have a closed form, we use the Monte Carlo method to obtain an approximate value of $\tau_{0}$ . First, we generate a sequence of values $\left\{(x_{j},z_{j})\;:\;j=1,\ldots ,N\right\}$ from f(x,z) for a sufficiently large *N*, and then we approximate $\tau_{0}$ by $\frac{1}{N}\sum_{\;j=1}^{N}P(Y=1|T=1,X=x_{j},Z=z_{j})-\frac{1}{N}\sum_{\;j=1}^{N}P(Y=1|T=0,X=x_{j},Z=z_{j})$ . In our numerical studies, we set N=5000 , yielding the true value $\tau_{0}$ to be 0.187 approximately.

### 4.2 Simulation results

Our objectives here are to (a) select important variables from the treatment model (3), (b) assess the performance of the proposed estimator of $\tau_{0}$ , and (c) demonstrate the effects of measurement error. When implementing the proposed method described in Section 3.1, we consider the following choices of relevant quantities. In Steps 3 and 5, we set C={0,0.25,0.50,0.75,1.0,1.25,1.50,1.75,2.0} and K=500 ; to evaluate how estimation results of $\tau_{0}$ may change, we use the quadratic, linear, or rational linear extrapolation function to approximate the true extrapolation function, as discussed by Carroll et al.^17^ ${}^{\left(\mathrm{Section}\;5.3.2\right)}$ . In Step 4, we set $V_{n}$ in (6) to be the identity matrix and take the SCAD penalty for (7); in comparison, we also consider the use of the LASSO penalty, which does not satisfy the requirement of “ $a_{n}\to 0$ as n→∞ ” in condition (C).

In contrast to variable selection for objective (a), we also examine the performance based on the full model without doing the variable selection. To compare the performance of the methods, we use both the $L_{1}$ and $L_{2}$ loss functions, respectively, given by $\sum_{k=0}^{p}|{\hat{\gamma }}_{k}-\gamma_{k}|\;\;\mathrm{and}\;\;\sum_{k=0}^{p}({\hat{\gamma }}_{k}-\gamma_{k}{)}^{2},$ where ${\hat{\gamma }}_{k}$ and $\gamma_{k}$ represent the *k*th component of $\hat{\gamma }$ and γ , respectively. In addition, we report the total number of selected variables and the number of falsely excluded important variables, denoted #S and #FN , respectively. Regarding objective (c) of demonstrating the impact of measurement error, we examine the naive approach, which disregards the difference between $X_{i}^{*}$ and $X_{i}$ in variable selection and estimation of $\tau_{0}$ .

To save space, here we report only the results for the cases with the treatment model specified as the logistic regression model and defer the results for the other two treatment models to the Supplemental material. Table 1 records the results for variable selection of the treatment model, and Table 2 reports finite sample biases (Bias), empirical standard errors (S.E.), root mean squared errors (RMSE), and coverage rates in percent (CR%) for 95% confidence intervals for $\tau_{0}$ obtained under the response Models 1 and 2.

**Table 1.** Simulation results: variable selection for the treatment model postulated by the logistic model.

		Proposed: quadratic extrapolation				Proposed: linear extrapolation				Proposed: rational linear extrapolation				Naive			
$\sigma_{e}^{2}$	Method	$L_{1}$ -loss	$L_{2}$ -loss	#S	#FN	$L_{1}$ -loss	$L_{2}$ -loss	#S	#FN	$L_{1}$ -loss	$L_{2}$ -loss	#S	#FN	$L_{1}$ -loss	$L_{2}$ -loss	#S	#FN
0.15	LASSO	0.322	0.013	18.363	0.000	0.405	0.028	18.557	0.000	0.395	0.023	18.530	0.000	2.528	0.468	22.190	0.000
	SCAD	0.313	0.011	17.256	0.000	0.353	0.022	17.477	0.000	0.349	0.020	17.309	0.000	2.489	0.447	22.167	0.000
	full	0.553	0.028	—	—	0.718	0.062	—	—	0.624	0.053	—	—	2.843	0.655	—	—
0.50	LASSO	0.337	0.015	18.570	0.000	0.423	0.033	18.593	0.000	0.414	0.029	18.554	0.000	2.607	0.474	22.231	0.000
	SCAD	0.326	0.014	17.368	0.000	0.388	0.029	17.536	0.000	0.380	0.027	17.388	0.000	2.538	0.456	22.184	0.000
	full	0.580	0.034	—	—	0.724	0.069	—	—	0.642	0.054	—	—	2.896	0.660	—	—
0.75	LASSO	0.349	0.018	18.615	0.000	0.440	0.037	18.673	0.000	0.423	0.034	18.650	0.000	2.634	0.486	22.325	0.000
	SCAD	0.342	0.017	17.381	0.000	0.421	0.034	17.589	0.000	0.411	0.032	17.430	0.000	2.597	0.475	22.249	0.000
	full	0.595	0.037	—	—	0.731	0.071	—	—	0.660	0.056	—	—	2.923	0.678	—	—
True *X*	LASSO	0.316	0.011	17.231	0.000	—	—	—	—	—	—	—	—	—	—	—	—
	SCAD	0.310	0.010	17.156	0.000	—	—	—	—	—	—	—	—	—	—	—	—
	full	0.536	0.025	—	—	—	—	—	—	—	—	—	—	—	—	—	—

LASSO: least absolute shrinkage and selection operator; SCAD: smoothly clipped absolute deviation. “Proposed” refers to the procedure in Section 3 using the surrogate $X_{i}^{*}$ together with other measurements; “Naive” represents the estimation procedure in Section 2 with $X_{i}$ replaced by $X_{i}^{*}$ ; “True *X*” denotes the estimation procedure in Section 2 using $X_{i}$ together with other measurements.

**Table 2.** Simulation results: estimation of ATE $\tau_{0}$ with the treatment model postulated by the logistic model.

			Proposed: quadratic extrapolation				Proposed: linear extrapolation				Proposed: rational linear extrapolation				Naive			
Model	$\sigma_{e}^{2}$	Method	Bias	S.E.	RMSE	CR%	Bias	S.E.	RMSE	CR%	Bias	S.E.	RMSE	CR%	Bias	S.E.	RMSE	CR%
1	0.15	LASSO	0.031	0.023	0.039	94.7	0.035	0.030	0.046	94.5	0.034	0.026	0.043	94.3	0.146	0.020	0.147	47.3
		SCAD	0.026	0.020	0.033	95.2	0.034	0.028	0.044	94.7	0.031	0.023	0.039	94.5	0.137	0.018	0.138	49.5
		full	0.058	0.026	0.064	88.4	0.064	0.035	0.073	86.3	0.061	0.031	0.068	87.8	0.159	0.024	0.161	42.6
	0.50	LASSO	0.033	0.025	0.041	94.7	0.039	0.033	0.051	94.4	0.035	0.029	0.045	94.1	0.155	0.022	0.157	43.4
		SCAD	0.028	0.023	0.036	95.0	0.037	0.030	0.048	94.5	0.034	0.028	0.044	94.3	0.149	0.020	0.150	45.2
		full	0.063	0.029	0.069	86.8	0.068	0.042	0.080	84.6	0.066	0.036	0.075	86.1	0.167	0.027	0.169	38.4
	0.75	LASSO	0.035	0.028	0.045	94.5	0.042	0.036	0.055	93.8	0.038	0.032	0.050	94.0	0.163	0.025	0.165	40.6
		SCAD	0.033	0.026	0.042	94.8	0.041	0.034	0.053	94.0	0.036	0.030	0.047	94.2	0.157	0.023	0.159	42.0
		full	0.069	0.032	0.076	83.7	0.071	0.045	0.084	82.4	0.069	0.038	0.079	86.0	0.173	0.029	0.175	34.2
	True *X*	LASSO	0.026	0.019	0.032	95.1	—	—	—	—	—	—	—	—	—	—	—	—
		SCAD	0.023	0.017	0.029	95.3	—	—	—	—	—	—	—	—	—	—	—	—
		full	0.053	0.024	0.058	91.3	—	—	—	—	—	—	—	—	—	—	—	—
2	0.15	LASSO	0.020	0.027	0.034	95.2	0.039	0.032	0.050	94.3	0.036	0.030	0.047	94.4	0.216	0.020	0.217	23.6
		SCAD	0.018	0.023	0.029	95.4	0.035	0.028	0.045	94.4	0.032	0.026	0.041	94.6	0.208	0.017	0.209	26.7
		full	0.063	0.036	0.073	86.5	0.071	0.042	0.082	85.1	0.068	0.039	0.078	86.7	0.256	0.030	0.258	18.5
	0.50	LASSO	0.024	0.029	0.038	95.0	0.042	0.034	0.054	94.0	0.040	0.032	0.051	94.1	0.223	0.023	0.224	20.1
		SCAD	0.022	0.025	0.033	95.2	0.039	0.030	0.049	94.1	0.037	0.030	0.048	94.3	0.216	0.019	0.217	22.4
		full	0.072	0.039	0.082	84.7	0.076	0.045	0.088	84.7	0.073	0.041	0.084	85.8	0.266	0.034	0.268	16.1
	0.75	LASSO	0.027	0.030	0.040	95.0	0.044	0.037	0.057	93.8	0.043	0.034	0.055	94.0	0.250	0.026	0.251	18.6
		SCAD	0.026	0.028	0.038	95.0	0.043	0.034	0.055	93.8	0.040	0.033	0.052	94.0	0.238	0.024	0.239	20.1
		full	0.079	0.043	0.090	82.6	0.084	0.047	0.096	84.1	0.082	0.044	0.093	84.7	0.307	0.035	0.309	11.4
	True *X*	LASSO	0.017	0.023	0.029	95.3	—	—	—	—	—	—	—	—	—	—	—	—
		SCAD	0.015	0.020	0.025	95.4	—	—	—	—	—	—	—	—	—	—	—	—
		full	0.063	0.030	0.070	91.1	—	—	—	—	—	—	—	—	—	—	—	—

ATE: average treatment effect; S.E.: standard error; RMSE: root mean square error; LASSO: least absolute shrinkage and selection operator; SCAD: smoothly clipped absolute deviation. CR%: coverage rates in percent. “Proposed” refers to the procedure in Section 3 using the surrogate $X_{i}^{*}$ together with other measurements, “Naive” represents the estimation procedure in Section 2 with $X_{i}$ replaced by $X_{i}^{*}$ , and “True *X*” denotes the estimation procedure in Section 2 using $X_{i}$ together with other measurements.

First, we examine the performance of the proposed method. Comparing the values of the $L_{1}$ and $L_{2}$ loss functions produced from the proposed method and the likelihood estimation method based on the full model, Table 1 shows the benefits of conducting variable selection for the treatment model (3). With the proposed method, using the quadratic extrapolation function tends to perform the best while the linear extrapolation function seems to perform the least well. The proposed method with the SCAD penalty inclines to slightly outperform the proposed method with the LASSO penalty. As expected, as the degree of measurement error increases, the proposed method produces increasing values of the $L_{1}$ and $L_{2}$ loss functions. Regarding the number (#S) of selected variables, the LASSO penalty tends to produce larger values than those obtained from using the SCAD, and both seem to be slightly larger than the true value.

Regarding the performance of estimating the ATE $\tau_{0}$ , Table 2 shows that the proposed method outperforms the method without excluding unimportant variables (i.e. under the full model). Estimation of $\tau_{0}$ without incorporating variable selection for building the treatment model (3) yields larger finite sample biases and standard errors than the proposed method, and the resulting coverage rates for 95% confidence intervals of $\tau_{0}$ deviate from the nominal level 95%. This suggests that imposing variable selection in the procedures of estimating $\tau_{0}$ is necessary.

With the three extrapolation functions, the proposed method generally performs well with small finite sample biases and good coverage rates for 95% confidence intervals of $\tau_{0}$ , and the performance with the quadratic extrapolation function tends to perform the best. Unsurprisingly, the performance of the proposed method deteriorates as the degree of measurement error increases. In implementing the proposed method, using the SCAD penalty yields slightly better results than using the LASSO penalty.

On the other hand, we inspect the results produced from the naive method, which ignores the measurement error effects. Evidently, as shown in Table 1, the naive method produces noticeable biases for the parameters of the treatment model (3) than the proposed method, though the number of falsely excluded important variables by the naive method, # FN, is near zero in all settings. Notably, Table 2 shows that the naive method produces unsatisfactory estimation results of $\tau_{0}$ with a lot larger finite sample biases yet smaller standard errors than those yielded from the proposed method, regardless of the outcome model, and thus, it leads to useless coverage rates for 95% confidence intervals of $\tau_{0}$ . Furthermore, RMSE values yielded from the naive method are larger than those of the proposed method under different settings, suggesting that the bias-variance trade-off of the proposed method is beneficial in contrast to that of the naive method. Similar patterns are also revealed in the tables included in Section A of the Supplemental material.

In summary, the simulation studies demonstrate the adverse effects of ignoring measurement error in the analysis as well as the importance of excluding irrelevant covariates when building the treatment model for calculating proper propensity scores. The studies confirm the satisfactory performance of the proposed estimator of $\tau_{0}$ in finite sample settings; the method with the SCAD penalty tends to slightly outperform the method with the LASSO penalty. As the proposed method is simulation-based, it may be time-consuming. However, its implementation is easy and can be built as a computerized-automatic program. Further, its applicability scope is broad in the sense that any parametric or semiparametric modeling for the treatment model (3) can be applied.

## 5 Analysis of NHANES I Epidemiologic Follow-up Study data

### 5.1 Data description

We apply the proposed method to analyze the data arising from the NHANES I Epidemiologic Follow-up Study (NHEFS), a national longitudinal study that was jointly initiated by the National Center for Health Statistics and the National Institute on Aging in collaboration with other agencies of the Public Health Service. The study was designed to investigate the relationship among clinical, nutritional, and behavioral factors. The NHEFS cohort includes participants of age 25 to 74 years who completed a medical examination at NHANES I in 1971–1975, and the first wave of data collection was conducted for all members from 1982 to 1984. The details can be found at https://wwwn.cdc.gov/nchs/nhanes/nhefs/#dfd.

In our analysis here, we consider a dataset of 1624 subjects, which is available at https://www.hsph.harvard.edu/miguel-hernan/causal-inference-book/. We are interested in understanding possible causal effects of smoking behavior on the weight change. Using the notation in Section 2, for an individual we let *T* represent the binary exposure variable for the smoking status (qsmk) (1 for quitting smoking or not smoking between the first questionnaire and 1982 and 0 otherwise). We want to estimate the average effect of *T*, $\tau_{0}=E(Y_{\left(1\right)})-E(Y_{\left(0\right)})$ , where $Y_{\left(1\right)}$ represents the weight an individual would have at the entry of the study if this person had been a nonsmoker or quitted smoking, and $Y_{\left(0\right)}$ represents the weight an individual would have at the entry of the study if this person had been a smoker. Let *Y* denote the actual weight in kilograms (wt) for an individual at the entry of the study.

Since the data are collected from the observational studies (and hence, smoking status cannot be randomized among the study subjects), directly calculating the difference for the sample average between the smokers and nonsmokers fails to yield a consistent estimator of $\tau_{0}$ . To control for the confounding effects and use (2) to estimate $\tau_{0}$ , we need first to build a suitable treatment model to calculate propensity scores.

The dataset we consider contains the following covariates, including systolic blood pressure (sbp, in millimeter of mercury (mmHg)), serum cholesterol (cholesterol, in mg/100), diastolic blood pressure (dbp, in mmHg), height in centimeters (ht), average tobacco price in the state of residence in 1982 (price82, in US dollars), age in 1971 (age, in years), sex (0 for male and 1 for female), use nerves medication (nerves, with 0 representing never and 1 otherwise), use high blood pressure medication (hbpmed, with 0 representing never use and 1 otherwise), and race (0 for white and 1 otherwise). Here each variable is standardized by subtracting its sample mean and then being divided by its sample standard deviation.

Blood pressure and cholesterol are error-prone, as discussed, respectively, by Bauldry et al.^29^ and Glasziou et al.^30^ As commented by Lebow and Rudd,^31^ ${}^{\left(p.\;168\right)}$ the price of tobacco is subject to measurement error due to respondents’ imprecise recall of their own expenditure or their unwillingness of reporting expenditures. Let *X* represent the vector of error-prone covariates including sbp ( $X_{1}$ ), dbp ( $X_{2}$ ), cholesterol ( $X_{3}$ ), and price82 ( $X_{4}$ ). Let *Z* denote the vector of error-free covariates including ht, age, sex, nerves, hbpmed, and race. Using the notation in Section 4.1, we have that $p_{x}=4$ , $p_{z}=1+6$ with 1 indicating the inclusion of the intercept in *Z*. We employ model (5) to describe the relationship between the observed surrogate measurement $X^{*}$ and the value of the true covariate *X*.

### 5.2 Analysis using external information

To estimate the covariance matrix in model (5), here we make use of external information of repeated measurements for sbp, dbp, cholesterol, and price82. Specifically, two repeated measurements, $X_{i1k}^{*}$ and $X_{i2k}^{*}$ , of the variables sbp ( $X_{i1}$ ) and dbp ( $X_{i2}$ ) for the same subjects are available at https://archive.ics.uci.edu/ml/datasets/Myocardial+infarction+complications, where i=574 and $k=1,\ldots ,n_{\left(1,2\right)}$ with $n_{\left(1,2\right)}=2$ . Three repeated measurements, $X_{i3k}$ , of the the variable cholesterol ( $X_{i3}$ ) for a group of people are posted at https://www.sheffield.ac.uk/mash/statistics/datasets, where i=18 and $k=1,\ldots ,n_{3}$ with $n_{3}=3$ . Eleven repeated measurements, $X_{i4k}$ , of the variable price82 ( $X_{i4}$ ) for the same individuals are available in the R package “**Ecdar**,” where i=48 and $k=1,\ldots ,n_{4}$ with $n_{4}=11$ .

With those repeated measurements, the estimated covariance matrix of $\Sigma_{e}$ is given by

$${\hat{\Sigma }}_{e}=\text{diag}\left({\hat{\Sigma }}_{12}^{*},{\hat{\sigma }}_{33}^{*2},{\hat{\sigma }}_{44}^{*2}\right),$$

where

$${\hat{\Sigma }}_{12}^{*}=\left(\begin{array}{cc} 0.499 & 0.434\\ 0.434 & 0.543 \end{array}\right),$$

${\hat{\sigma }}_{33}^{*2}=0.006$ , and ${\hat{\sigma }}_{44}^{*2}=0.150$ are, respectively, obtained from the three different data sources described above using the method of moments,

$$\frac{\sum_{i=1}^{n}\sum_{k=1}^{n_{j}}(X_{ijk}^{*}-{\bar{X}}_{ij}^{*})(X_{ijk}^{*}-{\bar{X}}_{ij}^{*}{)}^{⊤}}{\sum_{i=1}^{n}(n_{j}-1)}$$

for j=(1,2),3,4 , ${\bar{X}}_{ij}^{*}=n_{j}^{-1}\sum_{k=1}^{n_{j}}$ , and the independence of the $X_{ijk}^{*}$ is assumed for j=1,2,3,4 .

To implement the proposed method in Section 3.1, we consider three forms for the treatment model (3): the logistic, probit, and complementary log–log models; and we examine three extrapolation functions in Steps 3 and 5 in Section 3.1: quadratic, linear, and rational linear extrapolation functions. For variable selection, we use either the LASSO or SCAD penalty when implementing the proposed method based on (7). In comparison, we also examine the implementation based on the full model without doing variable selection like in Section 4, and call this method “full.” In the top two panels of Table 3, we report the estimates of the model parameters for the logistic regression treatment model obtained from different methods as well as the estimation results of $\tau_{0}$ , and defer the results for the other two treatment models to Section B in the Supplemental material.

**Table 3.** Analysis results of NHEFS data with propensity scores determined by the logistic model: external data are used to characterize the measurement error degree.

	Quadratic			Linear			RL		
Covariate	full	LASSO	SCAD	full	LASSO	SCAD	full	LASSO	SCAD
Intercept	−1.162	−1.098	−1.159	−1.145	−1.089	−1.150	−1.093	−1.049	−1.099
sbp	0.052	—	—	0.065	—	—	0.073	—	—
dbp	1.199	1.146	1.188	1.141	1.092	1.098	1.280	1.222	1.274
cholesterol	−0.005	—	—	−0.029	—	—	−0.013	—	—
price82	−1.268	−0.216	−0.272	−1.080	−0.024	−0.032	−1.120	−0.062	−0.066
ht	0.060	—	—	0.090	—	—	0.036	—	—
age	0.047	—	—	0.268	—	—	0.273	—	—
sex	0.040	—	—	0.029	—	—	0.028	—	—
nerves	−0.050	—	—	−0.034	—	—	−0.070	—	—
hbpmed	0.015	—	—	0.022	—	—	−0.004	—	—
race	−1.300	−0.236	−0.296	−1.276	−0.220	−0.280	−1.319	−0.274	−0.324
$\hat{\tau }$	1.313	3.256	3.477	1.478	3.345	3.500	1.600	3.587	3.744
$S.E.(\;\hat{\tau }\;)$	1.013	0.931	0.925	1.024	0.977	0.963	1.130	1.044	1.038
p-value	0.194	<0.001	<0.001	0.148	<0.001	<0.001	0.157	<0.001	<0.001
${\hat{\tau }}_{F}$	—	3.554	3.951	—	3.375	3.245	—	3.754	3.609
$S.E.(\;{\hat{\tau }}_{F})$	—	1.312	1.293	—	0.588	0.590	—	1.048	1.038
p-value	—	0.007	0.002	—	<0.001	<0.001	—	<0.001	<0.001

ATE: average treatment effect; LASSO: least absolute shrinkage and selection operator; SCAD: smoothly clipped absolute deviation. Headings “Quadratic”, “Linear”, and “RL” refer to the extrapolation function approximated by the quadratic, linear, and rational linear functions, respectively. The top panel reports the results of variable selection for the treatment model; the middle panel displays the estimation results of the ATE $\tau_{0}$ ; and the bottom panel shows the estimation results of the ATE $\tau_{0}$ by forcefully including “age” and “sex” to the selected variables to form the final treatment model to estimate $\tau_{0}$ .

Those results show that regardless of the model assumption for the treatment model and the extrapolation function form, both the proposed LASSO and SCAD methods suggest the same important covariates, dbp and race, for determining propensity scores. The estimates of $\tau_{0}$ produced by the proposed LASSO and SCAD method are closer to each other than to those obtained from the “full” method; and the former estimates are smaller than the latter ones. The standard errors produced from the proposed method with the SCAD penalty are the smallest, and they are closer to those produced from the proposed method with the LASSO penalty than those obtained from the “full” method.

Under all the settings, the estimates determined by the proposed LASSO or SCAD method have *p*-values <0.001 , revealing evidence of the exposure effects of *T* on affecting the average weight difference for nonsmokers and smokers. This revealing is in line with the results of Kaufman et al.^32^ On the contrary, if not excluding irrelevant covariates when determining propensity scores, the resulting *p*-values derived from the “full” method are all >0.05 , showing no evidence that the smoking status has effects on affecting individuals’ weights.

To reflect the application scenario where the assignment of the treatment must depend on certain covariates, a referee suggested to forcefully include some covariates to form the final treatment model, together with the selected variables obtained from Steps 1 to 4 in Section 3.1. To this end, here we take the covariates, age and sex, as those that must enter the final treatment model. Let $\left\{X_{Ii},Z_{Ii}\right\}$ denote the covariates selected from Steps 1 to 4 in Section 3.1. We then modify the fitting model (8) by replacing $\left\{X_{Ii},Z_{Ii}\right\}$ with $\left\{X_{Ii},Z_{Ii},age,sex\right\}$ , and re-run Step 5 in Section 3.1 to obtain an estimate of $\tau_{0}$ ; let ${\hat{\tau }}_{F}$ and S.E. $\left({\hat{\tau }}_{F}\right)$ denote the resulting estimate and the associated standard error, respectively. The results for the logistic treatment model are presented in the bottom panel of Table 3; and the results for the probit and complement log–log treatment models are summarized, respectively, at the bottom panels of Tables B1 and B2 in the Supplemental material.

### 5.3 Sensitivity analyses

Using external data helps us estimate the covariance matrix in model (5) as illustrated in Section 5.2. The validity of the analysis in Section 5.2 requires the comparability between the measurement error processes of the external data and the data we analyze, also called the *transportability* condition.^33^ As it is not apparent that this condition holds, here we further carry out sensitivity analyses to understand the impact of measurement error on estimation (e.g. Chen and Yi^27^ and Chen and Yi^34^).

Let $\Sigma_{X}$ and $\Sigma_{X^{*}}$ denote the covariance matrix of *X* and $X^{*}$ , respectively, and let $\sigma_{Xij}$ , $\sigma_{X^{*}ij}$ , and $\sigma_{eij}$ denote the (i,j) entry of $\Sigma_{X}$ , $\Sigma_{X^{*}}$ , and $\Sigma_{e}$ , respectively. Measurement error model (5) suggests that $\sigma_{Xij}$ is smaller than $\sigma_{X^{*}ij}$ for all *i* and *j*. To consider possible scenarios of measurement error, we calculate the sample covariance ${\hat{\Sigma }}_{X^{*}}$ of $\Sigma_{X^{*}}$ and set $\sigma_{Xij}$ as $\sigma_{Xij}=0.9{\hat{\sigma }}_{X^{*}ij}$ where ${\hat{\sigma }}_{X^{*}ij}$ is the (i,j) entry of ${\hat{\Sigma }}_{X^{*}}$ , and

$$\begin{array}{r} {\hat{\Sigma }}_{X^{*}}=\left(\begin{array}{cccc} 1.000 & 0.560 & 0.155 & -0.066\\ 0.560 & 1.000 & 0.052 & -0.026\\ 0.155 & 0.052 & 1.000 & 0.053\\ -0.066 & -0.026 & 0.053 & 1.000 \end{array}\right). \end{array}$$

To specify $\sigma_{eij}$ , we use the reliability ratio $R_{ij}=\sigma_{Xij}/\sigma_{X^{*}ij}=\sigma_{Xij}/(\sigma_{Xij}+\sigma_{\epsilon ij})$ to guide us:

(13) $$\sigma_{eij}=(R_{ij}^{-1}-1)\sigma_{Xij}.$$

For ease of exposition, we take $R_{ij}$ as a constant for all *i* and *j* and let *R* denote it. In our analysis, we specifically consider R=0.65,0.75 or 0.85 , and in the top two panels of Tables 4 to 6 we report the estimates of the parameters in the logistic treatment model as well as estimation results of $\tau_{0}$ . Other results for the probit and complementary log–log treatment models are included in Section B of the Supplemental material.

**Table 4.** Sensitivity analyses for NHEFS data with propensity scores determined by the logistic model: a quadratic extrapolation function is used.

	R=0.65			R=0.75			R=0.85		
Covariate	full	LASSO	SCAD	full	LASSO	SCAD	full	LASSO	SCAD
intercept	−1.292	−1.501	−1.161	−1.303	−1.191	−1.124	−1.792	−1.895	−1.570
sbp	0.626	—	—	0.236	—	—	0.152	—	—
dbp	2.427	1.915	2.510	3.301	2.575	3.347	3.212	2.758	3.434
cholesterol	−0.057	—	—	−0.197	—	—	−0.250	—	—
price82	−1.321	−0.530	−0.538	−1.087	−0.414	−0.406	−1.281	−0.384	−0.384
ht	0.011	—	—	0.011	—	—	0.011	—	—
age	0.020	—	—	0.022	—	—	0.024	—	—
sex	0.078	—	—	0.068	—	—	0.037	—	—
nerves	−0.235	—	—	−0.179	—	—	−0.190	—	—
hbpmed	0.023	—	—	0.022	—	—	0.013	—	—
race	−1.034	−0.243	−0.251	−1.028	−0.225	−0.217	−1.098	−0.201	−0.201
$\hat{\tau }$	1.012	3.155	3.617	1.497	3.631	3.713	1.424	3.672	3.286
$S.E.(\;\hat{\tau }\;)$	1.411	1.203	1.022	1.658	1.259	1.104	1.623	1.375	1.259
p-value	0.473	0.008	<0.001	0.366	0.003	<0.001	0.380	0.007	0.009
${\hat{\tau }}_{F}$	—	3.886	3.424	—	3.633	3.915	—	3.094	3.143
$S.E.(\;{\hat{\tau }}_{F})$	—	1.152	1.474	—	1.296	0.898	—	1.379	1.119
p-value	—	0.001	0.020	—	0.005	<0.001	—	0.024	0.005

ATE: average treatment effect; LASSO: least absolute shrinkage and selection operator; SCAD: smoothly clipped absolute deviation. The top panel reports the results of variable selection for the treatment model; the middle panel displays the estimation results of the ATE $\tau_{0}$ ; and the bottom panel shows the estimation results of the ATE $\tau_{0}$ by forcefully including “age” and “sex” to the selected variables to form the final treatment model to estimate $\tau_{0}$ .

**Table 5.** Sensitivity analyses for NHEFS data with propensity scores determined by the logistic model: a linear extrapolation function is used.

	R=0.65			R=0.75			R=0.85		
Covariate	full	LASSO	SCAD	full	LASSO	SCAD	full	LASSO	SCAD
intercept	−1.478	−1.031	−1.387	−1.956	−1.441	−1.866	−1.128	−1.648	−1.032
sbp	0.108	—	—	0.191	—	—	0.130	—	—
dbp	1.117	0.888	1.123	1.265	0.967	1.202	1.186	0.929	1.172
cholesterol	−0.016	—	—	−0.019	—	—	−0.052	—	—
price82	−0.598	−0.151	−0.169	−0.470	−0.145	−0.153	−0.428	−0.133	−0.146
ht	0.008	—	—	0.008	—	—	0.009	—	—
age	0.022	—	—	0.023	—	—	0.023	—	—
sex	0.049	—	—	0.072	—	—	0.066	—	—
nerves	−0.171	—	—	−0.153	—	—	−0.140	—	—
hbpmed	0.009	—	—	0.005	—	—	0.003	—	—
race	−0.881	−0.434	−0.493	−0.898	−0.383	−0.402	−0.900	−0.420	−0.451
$\hat{\tau }$	1.658	3.713	3.520	1.212	3.915	3.756	1.249	3.844	3.652
$S.E.(\;\hat{\tau }\;)$	1.155	1.088	1.128	1.612	1.014	0.997	1.374	1.245	1.224
p-value	0.265	0.001	0.002	0.452	<0.001	<0.001	0.363	0.001	0.003
${\hat{\tau }}_{F}$	—	3.523	3.501	—	3.467	3.263	—	3.055	2.977
$S.E.(\;{\hat{\tau }}_{F})$	—	1.216	1.215	—	0.883	0.900	—	1.050	1.048
p-value	—	0.004	0.004	—	<0.001	<0.001	—	0.004	0.003

ATE: average treatment effect; LASSO: least absolute shrinkage and selection operator; SCAD: SCAD: smoothly clipped absolute deviation. The top panel reports the results of variable selection for the treatment model; the middle panel displays the estimation results of the ATE $\tau_{0}$ ; and the bottom panel shows the estimation results of the ATE $\tau_{0}$ by forcefully including “age” and “sex” to the selected variables to form the final treatment model to estimate $\tau_{0}$ .

**Table 6.** Sensitivity analyses for NHEFS data with propensity scores determined by the logistic model: a rational linear extrapolation function is used.

	R=0.65			R=0.75			R=0.85		
Covariate	full	LASSO	SCAD	full	LASSO	SCAD	full	LASSO	SCAD
intercept	−1.163	−1.116	−1.170	−1.145	−1.092	−1.145	−1.149	−1.099	−1.156
sbp	0.069	—	—	0.035	—	—	0.062	—	—
dbp	1.295	1.234	1.288	1.277	1.224	1.277	1.208	1.152	1.187
cholesterol	−0.001	—	—	−0.008	—	—	−0.002	—	—
price82	−0.080	−0.033	−0.033	−0.103	−0.050	−0.050	−0.100	−0.050	−0.051
ht	0.100	—	—	0.091	—	—	0.093	—	—
age	0.271	—	—	0.260	—	—	0.260	—	—
sex	0.011	—	—	0.011	—	—	0.014	—	—
nerves	−0.070	—	—	−0.041	—	—	−0.067	—	—
hbpmed	0.012	—	—	0.016	—	—	0.015	—	—
race	−0.815	−0.268	−0.322	−0.827	−0.275	−0.328	−0.834	−0.254	−0.312
$\hat{\tau }$	1.236	3.188	3.433	0.955	3.209	3.107	1.436	3.139	3.650
$S.E.(\;\hat{\tau }\;)$	1.520	1.062	1.024	1.255	1.189	1.160	1.198	0.961	0.918
p-value	0.219	<0.001	<0.001	0.447	0.001	0.002	0.231	<0.001	<0.001
${\hat{\tau }}_{F}$	—	3.210	3.084	—	3.166	3.073	—	3.796	3.445
$S.E.(\;{\hat{\tau }}_{F})$	—	1.025	1.014	—	0.803	0.737	—	1.216	1.177
p-value	—	0.002	0.002	—	<0.001	<0.001	—	0.002	0.003

ATE: average treatment effect; LASSO: least absolute shrinkage and selection operator; SCAD: smoothly clipped absolute deviation. The top panel reports the results of variable selection for the treatment model; the middle panel displays the estimation results of the ATE $\tau_{0}$ ; and the bottom panel shows the estimation results of the ATE $\tau_{0}$ by forcefully including “age” and “sex” to the selected variables to form the final treatment model to estimate $\tau_{0}$ .

The estimation results are, in nature, consistent with the numerical results reported in Section 5.2. The variables dbp and race are selected to determine the propensity scores regardless of the values of *R*. For the estimation of $\tau_{0}$ , the proposed method with either the LASSO or SCAD penalty shows the significance effect of *T*, whereas the results obtained from the “full” model do not reveal such an effect.

Similar to Section 5.2, here we report sensitivity analyses results for ${\hat{\tau }}_{F}$ and $S.E.({\hat{\tau }}_{F})$ . The bottom panels of Tables 4 to 6 show the results for taking the treatment model as a logistic regression form. The results under the probit and complement log–log treatment models are placed in the bottom panels of Tables B3 to B8 in the Supplemental material.

## 6 Discussions and extensions

The inverse probability weighting estimation method and its variants have proved to be useful for estimating the average treatment effect in the causal inference framework. Despite the popularity of these methods, their applications are hindered by two critical conditions. The validity of those methods relies on the proper determination of propensity scores and the precise measurements of the confounders. In this article, we develop a simulation-based method by adapting the inverse probability weighting scheme to accommodate measurement error effects as well as variable selection for calculating propensity scores.

To highlight the idea, we present the proposed method by assuming the covariance matrix $\Sigma_{e}$ for model (5) to be given. This condition is needed only for Step 1 in Section 3. However, it is restrictive for applications. To circumvent the issues induced by unknown $\Sigma_{e}$ , we often need an additional data source to gain an understanding of the measurement error degree. If a validation sample having measurements for both $X_{i}$ and $X_{i}^{*}$ is available, $\Sigma_{e}$ can be estimated by fitting model (5) to the validation data. If a prior study on the same variables is available for the estimation of $\Sigma_{e}$ , one may use the estimated $\Sigma_{e}$ to implement Step 1 in Section 3. When repeated surrogate measurements are available, one may use them to estimate $\Sigma_{e}$ , as done in Section 5.2, or alternatively, one may modify Step 1 in Section 3 to get around the problem of unknown $\Sigma_{e}$ by applying the method of Devanarayan and Stefanski.^35^ In circumstances where no additional data sources are available, we often conduct sensitivity analyses to understand the impact of different degrees of measurement error on estimation of $\tau_{0}$ , as demonstrated in Section 5.3. Basically, we first specify a sequence of values for the covariance matrix $\Sigma_{e}$ to reflect possible scenarios of the measurement error process, and then we apply the proposed method to conduct the estimation of $\tau_{0}$ for each specified $\Sigma_{e}$ . Finally, we evaluate the sensitivity of estimation results to different magnitudes of measurement error. Such a study helps us understand how varying amounts of measurement error may affect estimation results.

The proposed method has a major limitation. As discussed in Section 3.1, the extrapolation functions in Steps 3 and 5 are unknown in applications and they can only be approximated by user-specified functions. Consistent with many studies about the SIMEX algorithm, we approximate the underlying true extrapolation functions by using one of the three functions, quadratic, linear, and rational linear functions, in our numerical studies. The simulation studies show that quadratic functions tend to outperform linear and rational linear functions, as in the same line with the numerical experience reported in the literature.^17^ ${}^{\left(\mathrm{Section}\;5.3.2\right)}$ Here we stress that the inference outcome can be sensitive to the choice of an approximating function. It is useful to develop a procedure to construct data-driven functions to well approximate the extrapolation functions to reach robust inference results. One possible approach is to start with a class of candidate functions, say F , then repeat the steps in Section 3.1 by taking each f∈F to approximate the extrapolation function, and let ${\hat{\tau }}_{f}$ and $v({\hat{\tau }}_{f})$ denote the resulting estimator and the associated variance, respectively. Then the optimal estimator of $\tau_{0}$ , ${\hat{\tau }}_{\;f_{0}}$ , is the estimator derived from using the function, $f_{0}={\mathrm{argmin}}_{\;f\in F}v({\hat{\tau }}_{f})$ , which minimizes $v({\hat{\tau }}_{f})$ . It will be interesting to carefully explore this idea through numerical studies and theoretical justifications by examining various classes F .

This research can be sharpened with several extensions which are outlined as follows. The proposed method focuses on the case where error-prone variables are all continuous. If error-prone variables are all discrete, one may modify the development here by replacing the SIMEX steps with the MC-SIMEX algorithm developed by Kuchenhoff et al.^36^ It is also interesting to generalize the development to accommodate a mix of error-contaminated discrete and continuous variables. One may adapt the *augmented simulation-extrapolation* method developed by Yi et al.^33^ and discussed by Chen and Yi^27^ and Zhang and Yi.^37^ To shed light on the extension, we consider the case with the vector $X_{i}$ only, where $X_{i}=(X_{Di},X_{Ci}^{T}{)}^{T}$ with $X_{Di}$ representing a binary variable and $X_{Ci}$ a subvector of continuous variables. Let $X_{Di}^{*}$ and $X_{Ci}^{*}$ denote the observed surrogate measurements of $X_{Di}$ and $X_{Ci}$ , respectively. Let $p_{i}=P(X_{Di}^{*}=0|X_{Di}=1,X_{Ci})$ and $q_{i}=P(X_{Di}^{*}=1|X_{Di}=0,X_{Ci})$ be the conditional misclassification probabilities, given $X_{i}$ , where the dependence of $p_{i}$ and $q_{i}$ on $X_{i}$ is suppressed in the notation.

One may employ logistic regression models to facilitate the dependence of the misclassification probabilities on the true variables $X_{i}$ :

$$\mathrm{logit}\;p_{i}=\alpha_{01}+\alpha_{x1}^{T}X_{Ci};\;\;\;\mathrm{logit}\;q_{i}=\alpha_{00}+\alpha_{x0}^{T}X_{Ci}$$

where $\alpha =(\alpha_{01},\alpha_{x1}^{T},\alpha_{00},\alpha_{x0}^{T}{)}^{T}$ is the vector of regression parameters.

For the error-prone continuous variables $X_{Ci}$ , we consider a model similar to (5):

$$X_{Ci}^{*}=X_{Ci}+e_{Ci},$$

where the error term $e_{Ci}$ is independent of $\left\{T_{i},X_{i},X_{Di}^{*},Y_{(0)i},Y_{(1)i}\right\}$ and follows $N(0,\Sigma_{Ce})$ with covariance matrix $\Sigma_{Ce}$ .

Now we write $S_{i}(\gamma ;X_{i})$ in (4) as $S_{i}(\gamma ;X_{Di},X_{Ci})$ to separately show the involvement of the discrete and continuous covariates. Define

$$\begin{array}{rl} S_{i}^{*}(\beta ;\gamma ,X_{Di}^{*},X_{Ci}) & =(1-p_{i}-q_{i}{)}^{-1}\\ & \;\times [(1-X_{Di}^{*})\{(1-p_{i})S_{i}(\beta ;\gamma ,0,X_{Ci})-q_{i}S_{i}(\beta ;\gamma ,1,X_{Ci})\}\\ & \;-X_{Di}^{*}\{p_{i}S_{i}(\beta ;\gamma ,0,X_{Ci})-(1-q_{i})S_{i}(\beta ;\gamma ,1,X_{Ci})\}] \end{array}$$

where $S_{i}(\beta ;\gamma ,r,X_{Ci})$ represents $S_{i}(\gamma ;X_{Di},X_{Ci})$ with $X_{Di}$ taking value *r*, and r=0,1 .

By Problem 2.10 of Yi^18^ ${}^{\left(p.83\right)}$ , $S_{i}^{*}(\beta ;\gamma ,X_{Di}^{*},X_{Ci})$ is an unbiased estimating function expressed in terms of the observed binary variable $X_{Di}^{*}$ , together with $X_{Ci}$ . To address the induced error effects of replacing $X_{Ci}$ with its surrogate $X_{Ci}^{*}$ , we may now employ the implementation procedure in Section 3.1 by replacing $S_{i}(\beta ;\gamma ,X_{Di},X_{Ci})$ with $S_{i}^{*}(\beta ;\gamma ,X_{Di}^{*},X_{Ci})$ in Step 2. Then the same steps carry through to obtain an estimator of $\tau_{0}$ with mismeasurement error effects in both $X_{Di}$ and $X_{Ci}$ accounted for.

For a more general case with multiple binary variables or categorical variables subject to measurement error, one may use the same ideas to develop an inference procedure by introducing a misclassification matrix and further modifying the construction of the function $S_{i}^{*}(\beta ;\gamma ,X_{Di}^{*},X_{Ci})$ with more involved notation. A careful study is warranted for working out technical details.

In Step 4 of the implementation procedure in Section 3.1, a common penalty function $p_{\lambda }(\cdot )$ is used for the components in γ to construct the penalized quadratic loss function (6). This practice essentially treats all the pre-treatment variables of $W_{i}$ in model (3) equally the same. In applications, with the subject matter information, we may need to include certain pre-treatment variables when building the treatment model. In this case, the formulation of the penalized quadratic loss function (6) can be modified by not imposing a penalty on the parameters corresponding to such variables. Alternatively, one may first implement the present development to do variable selection and then add the must-to-be-included variables (if not being selected) to build the final treatment model, together with those selected variables. It is useful to explore these two strategies in depth.

Our development roots in the use of a parametric model (3) to describe propensity scores. This consideration is primarily driven by the attractive features of parametric modeling. Parametric approaches are more effective than non-parametric methods in handling data with a large dimension, and they allow us to deal with measurement error effects using existing methods. Further, the asymptotic result of the resulting estimator can be established, which enables us to carry out statistical inference rigorously. While having these advantages, the parametric modeling scheme is vulnerable to model misspecification. To ameliorate this, machine learning methods have been employed to characterize propensity scores, which include classification and regression trees (e.g. Lee et al.^38^), neural networks and support vector machines (e.g. Westreich et al.^39^), and cross-fit estimators (e.g. Zivich and Breskin^40^). It is interesting to extend our development by using those machine learning methods to delineate the treatment model. One needs to carefully investigate typical issues related to machine learning methods, such as the lack of transparent interpretation or the nature of “black-box” of the algorithms, and the bias-and-variance trade-off.

## Supplemental Material

sj-pdf-1-smm-10.1177_09622802221146308 - Supplemental material for Estimation of the average treatment effect with variable selection and measurement error simultaneously addressed for potential confounders Click here for additional data file. Supplemental material, sj-pdf-1-smm-10.1177_09622802221146308 for Estimation of the average treatment effect with variable selection and measurement error simultaneously addressed for potential confounders by Grace Y. Yi and Li-Pang Chen in Statistical Methods in Medical Research

## Appendix: Proof of Theorem 3.2

Let $\gamma_{I}$ denote the parameters for the selected treatment model (8) and let $\phi (\gamma_{I};T_{i},X_{Ii},Z_{Ii})$ denote the score function of $\gamma_{I}$ derived from model (8). Let $\theta =(\gamma_{I}^{T},\tau {)}^{T}$ denote the parameters and let $\theta_{0}=(\gamma_{I0}^{T},\tau_{0}{)}^{T}$ denote their true values. Let $\hat{\theta }=({\hat{\gamma }}_{I}^{T},\hat{\tau }{)}^{T}$ denote the estimator of θ , referred in Theorems 3.1 and 3.2.

Define

(14)
$$U_{i}(\theta ;Y_{i},T_{i},X_{Ii},Z_{Ii})=\left(\begin{array}{c} \phi (\gamma ;T_{i},X_{Ii},Z_{Ii})\\ \frac{T_{i}Y_{i}}{\pi_{i}}-\frac{(1-T_{i})Y_{i}}{1-\pi_{i}}-\tau \end{array}\right).$$

It is readily shown that $U_{i}(\theta ;Y_{i},T_{i},X_{Ii},Z_{Ii})$ is an unbiased estimating function of θ , i.e.,

$$E\{U_{i}(\theta ;Y_{i},T_{i},X_{Ii},Z_{Ii})\}=0$$

where the expectation is evaluated with respect to the joint distribution for the associated random variables.

Now we discuss the asymptotic distribution for the estimator $\hat{\theta }$ for θ by modifying the discussions of Yi et al.^26^ and Carroll et al.^28^ For each *k* and ψ , define $U_{i}^{*}(\theta ;k,\psi )=U_{i}(\theta ;Y_{i},T_{i},X_{Ii}^{*}(k,\psi ),Z_{Ii})$ , where $X_{Ii}^{*}(k,\psi )$ is the subvector of $X_{i}^{*}(k,\psi )$ corresponding to the subvector $X_{Ii}^{*}$ of $X_{i}^{*}$ . Since for a given ψ , the $X_{Ii}^{*}(k,\psi )$ are independent for k=1,…,K , so the solutions of $E[U_{i}^{*}(\theta ;k,\psi )]=0$ are free of the values of *k*, where the expectation is evaluated under the distribution of the associated variables. Assuming that the equation has a unique solution, we let θ(ψ) denote such a solution.

Assuming that for each *k* and ψ ,

(15)
$$\sum_{i=1}^{n}U_{i}^{*}(\theta ;k,\psi )=0$$

has a unique solution, and let $\hat{\theta }(k,\psi )$ denote such a solution. Then applying Theorem 1 of Yi and Reid^41^ gives

$$\hat{\theta }(k,\psi )\overset{p}{\to }\theta (\psi )\;\;\;\mathrm{as}\;n\to \infty .$$

Applying the Taylor series expansion to (15) gives that

(16)
$$\sqrt{n}\{\hat{\theta }(k,\psi )-\theta (\psi )\}=-{\sqrt{n}}^{-1}\sum_{i=1}^{n}[\Gamma (\psi ){]}^{-1}U_{i}^{*}(\theta (\psi );k,\psi )+o_{p}(1)$$

where $\Gamma (\psi )=E[(\partial /\partial \theta )U_{i}^{*T}(\theta (\psi );k,\psi )]$ .

Let

$$\hat{\theta }(\psi )=\frac{1}{K}\sum_{k=1}^{K}\hat{\theta }(k,\psi )\;\mathrm{and}\;V_{i}(\psi )=\frac{1}{K}\sum_{k=1}^{K}[\Gamma (\psi ){]}^{-1}U_{i}^{*}(\theta (\psi );k,\psi ).$$

Thus, summing (16) over *k* and then dividing by *K* leads to

(17)
$$\sqrt{n}\{\hat{\theta }(\psi )-\theta (\psi )\}=-{\sqrt{n}}^{-1}\sum_{i=1}^{n}V_{i}(\psi )+o_{p}(1)\;\;\mathrm{for}\;\psi \in C.$$

Now we examine the extrapolation step for obtaining the SIMEX estimator $\hat{\theta }$ . Let *d* be the dimension of θ . Suppose the exact extrapolation function is known, that is, there is a known d×1 vector h(⋅) of functions of *m*-dimensional arguments such that the SIMEX estimator can be written as

$$\hat{\theta }=h(\hat{\theta }(\psi_{1}),\ldots ,\hat{\theta }(\psi_{M}))$$

and the true parameter value $\theta_{0}$ is related to $\left\{\theta (\psi_{1}),\ldots ,\theta (\psi_{M})\right\}$ by

$$\theta_{0}=h(\theta (\psi_{1}),\ldots ,\theta (\psi_{M})).$$

For j=1,…,M , let ${\dot{h}}_{j}=\partial h(\theta (\psi_{1}),\ldots ,\theta (\psi_{M}))/\partial \theta^{T}(\psi_{j})$ . Then applying the Taylor series expansion to $h(\hat{\theta }(\psi_{1}),\ldots ,\hat{\theta }(\psi_{M}))$ gives

$$h(\hat{\theta }(\psi_{1}),\ldots ,\hat{\theta }(\psi_{M}))=h(\theta (\psi_{1}),\ldots ,\theta (\psi_{M}))+\sum_{\;j=1}^{M}{\dot{h}}_{j}\times (\hat{\theta }(\psi_{j})-\theta (\psi_{j}))+o_{p}({\sqrt{n}}^{-1}),$$

thus

$$\sqrt{n}(\hat{\theta }-\theta_{0})=\sum_{\;j=1}^{M}{\dot{h}}_{j}\times \sqrt{n}(\hat{\theta }(\psi_{j})-\theta (\psi_{j}))+o_{p}(1).$$

Define $H_{i}=\sum_{\;j=1}^{M}{\dot{h}}_{j}\times V_{i}(\psi_{j})$ , then by (17), we have

(18)
$$\sqrt{n}(\hat{\theta }-\theta_{0})=-{\sqrt{n}}^{-1}\sum_{i=1}^{n}H_{i}+o_{p}(1).$$

Then applying the Central Limit Theorem to (18) gives

(19)
$$\sqrt{n}(\hat{\theta }-\theta_{0})\overset{p}{\to }N(0,\Sigma_{SIM})\;\;\;\mathrm{as}\;\;n\to \infty ,$$

where $\Sigma_{SIM}=\mathrm{var}(H_{i})$ . Let $v(\tau_{0})$ denote the right-lower corner element of $\Sigma_{SIM}$ . Then (19) yields that $\sqrt{n}(\hat{\tau }-\tau_{0})$ has an asymptotic normal distribution with mean zero and variance $v(\gamma_{0})$ .
