# Supplementary material for: Estimation of the average treatment effect with variable selection and measurement error simultaneously addressed for potential confounders
Source: Stat Methods Med Res. 2023 Jan 24;32(4):691–711. doi: 10.1177/09622802221146308 (PMC10119903; doi:10.1177/09622802221146308)
Supplement: sj-pdf-1-smm-10.1177_09622802221146308 - Supplemental material for Estimation of the average treatment effect with variable selection and measurement error simultaneously addressed for potential confounders [file sj-pdf-1-smm-10.1177_09622802221146308.pdf]

Supplementary Materials for
"Estimation of the Average Treatment Effect with Variable Selection and Measurement Error Simultaneously Addressed for Potential Confounders"

Grace Y. Yi* and Li-Pang Chen**

*University of Western Ontario, London, Ontario, Canada N6A 5B7
email: gyi5@uwo.ca; corresponding author
**National Chengchi University, Taipei, Taiwan
email: lchen723@nccu.edu.tw

The supplementary material contains additional numerical results for the manuscript, entitled "Estimation of the Average Treatment Effect with Variable Selection and Measurement Error Simultaneously Addressed for Potential Confounders", co-authored by Grace. Y. Yi and Li-Pang Chen.

A Additional Simulation Results

This appendix presents additional simulation results for settings where the treatment model is postulated by the probit regression or the complementary log-log regression model. When implementing the proposed method, we respectively use the quadratic, linear, and rational linear functions to approximate the extrapolation function. Tables A1 and A2 report the variable selection results for the treatment model, respectively, postulated by the probit and complementary log-log models. Tables A3 and A4 summarize the estimation results for ATE τ_0 under the two outcome models, where the treatment model is characterized by the probit and complementary log-log models, respectively.

Table A1: Simulation results: variable selection for the treatment model postulated by the probit model. "Proposed" refers to the procedure in Section 3 using the surrogate X_i^* together with other measurements, "Naive" represents the estimation procedure in Section 2 with X_i replaced by X_i^*, and "True X" denotes the estimation procedure in Section 2 using X_i together with other measurements.

σ_e^2	Method	Proposed: quadratic extrapolation				Proposed: linear extrapolation				Proposed: rational linear extrapolation				Naive			
		L_1-loss	L_2-loss	#S	#FN	L_1-loss	L_2-loss	#S	#FN	L_1-loss	L_2-loss	#S	#FN	L_1-loss	L_2-loss	#S	#FN
0.15	Lasso	0.330	0.014	18.340	0.000	0.384	0.021	18.711	0.000	0.382	0.020	18.532	0.000	2.849	0.488	23.711	0.000
	SCAD	0.315	0.012	17.503	0.000	0.366	0.020	17.615	0.000	0.353	0.020	17.588	0.000	2.615	0.453	22.565	0.000
	full	0.444	0.027	–	–	0.622	0.050	–	–	0.618	0.049	–	–	3.266	0.522	–	–
0.50	Lasso	0.345	0.016	18.378	0.000	0.398	0.025	18.789	0.000	0.389	0.022	18.630	0.000	2.978	0.497	23.833	0.000
	SCAD	0.329	0.015	17.617	0.000	0.373	0.021	17.660	0.000	0.362	0.020	17.621	0.000	2.766	0.478	22.733	0.000
	full	0.478	0.036	–	–	0.637	0.053	–	–	0.624	0.050	–	–	3.423	0.578	–	–
0.75	Lasso	0.353	0.019	18.397	0.000	0.405	0.027	18.935	0.000	0.394	0.024	18.744	0.000	3.017	0.508	24.110	0.000
	SCAD	0.348	0.018	17.801	0.000	0.388	0.024	17.886	0.000	0.374	0.022	17.863	0.000	2.914	0.482	22.867	0.000
	full	0.508	0.044	–	–	0.641	0.057	–	–	0.636	0.053	–	–	3.593	0.588	–	–
True X	Lasso	0.301	0.008	17.869	0.000	–	–	–	–	–	–	–	–	–	–	–	–
	SCAD	0.224	0.004	17.230	0.000	–	–	–	–	–	–	–	–	–	–	–	–
	full	0.398	0.016	–	–	–	–	–	–	–	–	–	–	–	–	–	–

Table A2: Simulation results: variable selection for the treatment model postulated by the complement log-log model. "Proposed" refers to the procedure in Section 3 using the surrogate X_i^* together with other measurements, "Naive" represents the estimation procedure in Section 2 with X_i replaced by X_i^*, and "True X" denotes the estimation procedure in Section 2 using X_i together with other measurements.

σ_e^2	Method	Proposed: quadratic extrapolation				Proposed: linear extrapolation				Proposed: rational linear extrapolation				Naive			
		L_1-loss	L_2-loss	#S	#FN	L_1-loss	L_2-loss	#S	#FN	L_1-loss	L_2-loss	#S	#FN	L_1-loss	L_2-loss	#S	#FN
0.15	Lasso	0.326	0.013	18.123	0.000	0.402	0.024	18.430	0.000	0.388	0.022	18.369	0.000	2.778	0.475	24.567	0.000
	SCAD	0.317	0.012	17.300	0.000	0.389	0.020	17.613	0.000	0.360	0.021	17.604	0.000	2.750	0.466	23.311	0.000
	full	0.411	0.025	–	–	0.506	0.042	–	–	0.466	0.036	–	–	3.378	0.565	–	–
0.50	Lasso	0.331	0.015	18.204	0.000	0.418	0.030	18.487	0.000	0.393	0.022	18.380	0.000	2.886	0.503	24.711	0.000
	SCAD	0.324	0.013	17.319	0.000	0.396	0.028	17.660	0.000	0.371	0.021	17.635	0.000	2.844	0.489	23.504	0.000
	full	0.440	0.033	–	–	0.510	0.044	–	–	0.471	0.043	–	–	3.416	0.597	–	–
0.75	Lasso	0.339	0.016	18.285	0.000	0.439	0.036	18.511	0.000	0.414	0.024	18.407	0.000	2.961	0.522	25.123	0.000
	SCAD	0.332	0.015	17.334	0.000	0.420	0.032	17.684	0.000	0.390	0.023	17.651	0.000	2.933	0.516	24.289	0.000
	full	0.462	0.039	–	–	0.519	0.047	–	–	0.495	0.045	–	–	3.526	0.613	–	–
True X	Lasso	0.316	0.013	17.898	0.000	–	–	–	–	–	–	–	–	–	–	–	–
	SCAD	0.296	0.011	17.218	0.000	–	–	–	–	–	–	–	–	–	–	–	–
	full	0.381	0.021	–	–	–	–	–	–	–	–	–	–	–	–	–	–

Table A3: Simulation results: estimation of ATE τ_0 with the treatment model postulated by the probit model. "Proposed" refers to the procedure in Section 3 using the surrogate X_i^* together with other measurements, "Naive" represents the estimation procedure in Section 2 with X_i replaced by X_i^*, and "True X" denotes the estimation procedure in Section 2 using X_i together with other measurements.

Model	σ_e^2	Method	Proposed: quadratic extrapolation				Proposed: linear extrapolation				Proposed: rational linear extrapolation				Naive			
			Bias	S.E.	RMSE	CR%	Bias	S.E.	RMSE	CR%	Bias	S.E.	RMSE	CR%	Bias	S.E.	RMSE	CR%
1	0.15	Lasso	0.030	0.020	0.036	94.7	0.033	0.028	0.043	94.6	0.033	0.026	0.042	94.4	0.135	0.017	0.136	50.7
		SCAD	0.027	0.018	0.032	95.3	0.032	0.025	0.041	94.6	0.031	0.025	0.040	94.7	0.130	0.016	0.131	53.9
		full	0.050	0.026	0.056	90.1	0.063	0.031	0.070	88.7	0.058	0.030	0.065	88.9	0.159	0.022	0.161	40.3
	0.50	Lasso	0.033	0.024	0.041	94.4	0.036	0.031	0.048	94.4	0.035	0.027	0.044	94.3	0.142	0.020	0.143	48.6
		SCAD	0.030	0.021	0.037	95.0	0.034	0.027	0.043	94.4	0.033	0.025	0.041	94.4	0.138	0.018	0.139	51.3
		full	0.054	0.030	0.062	90.0	0.065	0.034	0.073	87.3	0.060	0.032	0.068	87.5	0.164	0.026	0.166	37.8
	0.75	Lasso	0.034	0.026	0.043	94.4	0.038	0.033	0.050	94.4	0.036	0.028	0.046	94.2	0.150	0.022	0.152	42.0
		SCAD	0.033	0.023	0.040	95.0	0.035	0.028	0.045	94.2	0.034	0.026	0.043	94.3	0.144	0.020	0.145	47.5
		full	0.058	0.033	0.067	88.7	0.068	0.035	0.076	85.6	0.064	0.035	0.073	86.4	0.171	0.029	0.173	31.1
	True X	Lasso	0.026	0.018	0.032	95.2	–	–	–	–	–	–	–	–	–	–	–	–
		SCAD	0.023	0.015	0.027	95.4	–	–	–	–	–	–	–	–	–	–	–	–
		full	0.047	0.024	0.053	91.1	–	–	–	–	–	–	–	–	–	–	–	–
2	0.15	Lasso	0.022	0.025	0.033	95.1	0.030	0.031	0.043	94.4	0.029	0.029	0.041	94.6	0.208	0.019	0.209	25.5
		SCAD	0.021	0.022	0.030	95.2	0.027	0.029	0.040	94.7	0.025	0.026	0.036	95.0	0.203	0.018	0.204	28.2
		full	0.071	0.032	0.078	84.7	0.078	0.036	0.086	84.0	0.075	0.033	0.082	84.3	0.247	0.028	0.249	20.3
	0.50	Lasso	0.026	0.028	0.038	94.8	0.034	0.033	0.047	94.0	0.032	0.032	0.045	94.3	0.221	0.023	0.222	21.2
		SCAD	0.024	0.025	0.035	95.1	0.030	0.030	0.042	94.4	0.028	0.030	0.041	94.7	0.215	0.021	0.216	25.5
		full	0.078	0.036	0.086	84.0	0.085	0.041	0.094	83.4	0.082	0.038	0.090	83.8	0.260	0.032	0.262	14.4
	0.75	Lasso	0.029	0.029	0.041	94.6	0.037	0.036	0.052	93.7	0.034	0.033	0.047	94.0	0.230	0.025	0.231	18.6
		SCAD	0.027	0.028	0.039	95.0	0.035	0.034	0.049	94.1	0.032	0.032	0.045	94.5	0.226	0.024	0.227	20.4
		full	0.088	0.039	0.096	78.4	0.093	0.044	0.103	83.1	0.090	0.041	0.099	83.5	0.281	0.034	0.283	11.6
	True X	Lasso	0.018	0.020	0.027	95.2	–	–	–	–	–	–	–	–	–	–	–	–
		SCAD	0.018	0.020	0.027	95.3	–	–	–	–	–	–	–	–	–	–	–	–
		full	0.060	0.031	0.068	90.2	–	–	–	–	–	–	–	–	–	–	–	–

Table A4: Simulation results: estimation of ATE τ_0 with the treatment model postulated by the complement log-log model. "Proposed" refers to the procedure in Section 3 using the surrogate X_i^* together with other measurements, "Naive" represents the estimation procedure in Section 2 with X_i replaced by X_i^*, and "True X" denotes the estimation procedure in Section 2 using X_i together with other measurements.

Model	σ_e^2	Method	Proposed: quadratic extrapolation				Proposed: linear extrapolation				Proposed: rational linear extrapolation				Naive			
			Bias	S.E.	RMSE	CR%	Bias	S.E.	RMSE	CR%	Bias	S.E.	RMSE	CR%	Bias	S.E.	RMSE	CR%
1	0.15	Lasso	0.028	0.023	0.036	95.0	0.039	0.026	0.047	94.2	0.036	0.025	0.044	94.5	0.144	0.016	0.145	46.3
		SCAD	0.022	0.020	0.030	95.3	0.035	0.024	0.042	94.4	0.032	0.022	0.039	94.6	0.139	0.013	0.140	48.2
		full	0.066	0.029	0.072	88.4	0.071	0.033	0.078	86.3	0.069	0.031	0.076	86.7	0.160	0.024	0.162	23.7
	0.50	Lasso	0.031	0.027	0.041	95.0	0.041	0.029	0.050	94.2	0.038	0.026	0.046	94.3	0.153	0.018	0.154	40.5
		SCAD	0.027	0.023	0.035	95.1	0.035	0.026	0.044	94.3	0.035	0.024	0.042	94.3	0.146	0.016	0.147	42.7
		full	0.067	0.034	0.075	88.4	0.074	0.036	0.082	85.7	0.071	0.033	0.078	86.0	0.178	0.027	0.180	17.1
	0.75	Lasso	0.036	0.029	0.046	94.8	0.045	0.033	0.056	94.0	0.040	0.028	0.049	94.0	0.164	0.023	0.166	34.2
		SCAD	0.031	0.026	0.040	95.0	0.040	0.031	0.051	94.1	0.038	0.027	0.047	94.2	0.158	0.020	0.159	38.0
		full	0.070	0.034	0.078	87.5	0.077	0.038	0.086	84.4	0.074	0.036	0.082	85.3	0.184	0.032	0.187	15.5
	True X	Lasso	0.023	0.020	0.030	95.3	–	–	–	–	–	–	–	–	–	–	–	–
		SCAD	0.019	0.019	0.027	95.3	–	–	–	–	–	–	–	–	–	–	–	–
		full	0.060	0.025	0.065	90.7	–	–	–	–	–	–	–	–	–	–	–	–
2	0.15	Lasso	0.020	0.023	0.030	95.3	0.026	0.030	0.040	94.5	0.022	0.027	0.035	95.0	0.211	0.018	0.212	23.6
		SCAD	0.019	0.021	0.028	95.3	0.023	0.028	0.036	94.5	0.020	0.025	0.032	95.0	0.208	0.016	0.209	24.5
		full	0.050	0.029	0.058	87.9	0.062	0.034	0.071	86.7	0.060	0.032	0.068	87.2	0.238	0.024	0.239	18.4
	0.50	Lasso	0.023	0.024	0.033	95.1	0.031	0.033	0.045	94.0	0.029	0.031	0.042	94.4	0.220	0.018	0.221	19.4
		SCAD	0.022	0.023	0.032	95.1	0.029	0.031	0.042	94.2	0.026	0.028	0.038	94.6	0.218	0.018	0.219	21.7
		full	0.056	0.033	0.065	86.4	0.069	0.037	0.078	85.8	0.066	0.036	0.075	86.0	0.245	0.026	0.246	14.5
	0.75	Lasso	0.025	0.027	0.037	94.9	0.034	0.035	0.049	93.7	0.032	0.033	0.046	94.1	0.231	0.020	0.0232	16.8
		SCAD	0.023	0.026	0.035	95.0	0.031	0.033	0.045	93.8	0.029	0.030	0.042	94.2	0.227	0.020	0.228	18.1
		full	0.059	0.035	0.069	86.0	0.075	0.040	0.085	85.3	0.071	0.039	0.081	85.6	0.256	0.029	0.258	12.8
	True X	Lasso	0.016	0.020	0.026	95.3	–	–	–	–	–	–	–	–	–	–	–	–
		SCAD	0.015	0.020	0.025	95.5	–	–	–	–	–	–	–	–	–	–	–	–
		full	0.044	0.026	0.051	91.6	–	–	–	–	–	–	–	–	–	–	–	–

B Additional Analyses of NHEFS Data

This appendix presents additional analyses of the NHEFS data, where the treatment model is descirbed by the probit or complementary log-log regression model. To implement the proposed method, we respectively use the quadratic, linear, and rational linear functions to approximate the extrapolation function.

With the external data in Section 5.2, we report the analysis results in Tables B1 and B2, where the treatment model is respectively postulated by the probit and complementary log-log models. For sensitivity analyses in Section 5.3, we display the results in Tables B3-B5 report the results for the setting with treatment model taken as the probit model, and we include in Tables B6-B8 for the case with the treatment model specified as the complement log-log model.

Table B1: Analysis results of NHEFS data with propensity scores determined by the probit model: external data are used to characterize the measurement error degree. Headings "Quadratic", "Linear", and "RL" refer to that the extrapolation function is approximated by the quadratic, linear and rational linear functions, respectively. The top panel reports the results of variable selection for the treatment model; the middle panel displays the estimation results of the ATE τ_0; and the bottom panel shows the estimation results of the ATE τ_0 by forcefully including "`age`" and "`sex`" to the selected variables to form the final treatment model to estimate τ_0.

Covariate	Quadratic			Linear			RL		
	full	LASSO	SCAD	full	LASSO	SCAD	full	LASSO	SCAD
intercept	-0.712	-0.675	-0.708	-0.686	-0.645	-0.679	-0.692	-0.654	-0.691
sbp	0.022	–	–	0.005	–	–	0.048	–	–
dbp	1.164	1.135	1.169	1.101	1.078	1.097	1.152	1.114	1.154
cholesterol	-0.021	–	–	-0.004	–	–	-0.021	–	–
price82	-1.034	-1.025	-1.025	-1.049	-1.009	-1.011	-1.035	-1.016	-1.021
ht	0.053	–	–	0.044	–	–	0.054	–	–
age	0.163	–	–	0.171	–	–	0.155	–	–
sex	0.007	–	–	0.011	–	–	0.016	–	–
nerves	-0.022	–	–	-0.010	–	–	-0.038	–	–
hbpmed	0.044	–	–	0.032	–	–	0.030	–	–
race	-0.877	-0.639	-0.672	-0.873	-0.632	-0.566	-0.889	-0.750	-0.688
$\widehat{\tau}$	1.259	3.647	3.551	1.277	3.873	3.781	1.337	3.803	3.860
S.E. $(\widehat{\tau})$	1.139	1.001	0.976	0.759	0.658	0.653	0.996	0.912	0.777
p-value	0.269	< 0.001	< 0.001	0.156	< 0.001	< 0.001	0.179	< 0.001	< 0.001
$\widehat{\tau}_{\mathrm{F}}$	–	3.777	3.722	–	3.199	3.137	–	3.861	3.882
S.E. $(\widehat{\tau}_{\mathrm{F}})$	–	1.145	1.122	–	1.247	1.250	–	1.141	1.092
p-value	–	0.001	0.001	–	0.010	0.010	–	< 0.001	< 0.001

Table B2: Analysis results of NHEFS data with propensity scores determined by the complement log-log model: external data are used to characterize the measurement error degree. Headings "Quadratic", "Linear", and "RL" refer to that the extrapolation function is approximated by the quadratic, linear and rational linear functions, respectively. The top panel reports the results of variable selection for the treatment model; the middle panel displays the estimation results of the ATE τ_0; and the bottom panel shows the estimation results of the ATE τ_0 by forcefully including "`age`" and "`sex`" to the selected variables to form the final treatment model to estimate τ_0.

Covariate	Quadratic			Linear			RL		
	full	LASSO	SCAD	full	LASSO	SCAD	full	LASSO	SCAD
intercept	-1.327	-1.266	-1.332	-1.327	-1.267	-1.343	-1.360	-1.300	-1.404
sbp	0.051	–	–	0.019	–	–	0.133	–	–
dbp	1.233	0.972	0.914	1.150	0.888	0.876	1.204	0.803	0.810
cholesterol	-0.009	–	–	-0.020	–	–	-0.004	–	–
price82	-1.078	-0.818	-0.821	-1.053	-0.843	-0.808	-1.050	-0.845	-0.831
ht	0.061	–	–	0.073	–	–	0.092	–	–
age	0.227	–	–	0.213	–	–	0.227	–	–
sex	0.014	–	–	0.004	–	–	0.041	–	–
nerves	-0.057	–	–	-0.044	–	–	-0.059	–	–
hbpmed	0.006	–	–	0.002	–	–	0.016	–	–
race	-0.818	-0.258	-0.324	-0.843	-0.283	-0.359	-0.806	-0.246	-0.351
$\widehat{\tau}$	1.586	3.202	2.902	1.386	3.328	3.229	1.264	3.930	3.848
S.E. $(\widehat{\tau})$	0.925	0.816	0.815	1.137	0.980	0.925	1.010	0.998	0.897
p-value	0.526	< 0.001	< 0.001	0.223	< 0.001	< 0.001	0.159	< 0.001	< 0.001
$\widehat{\tau}_{\mathrm{F}}$	–	4.156	4.089	–	4.027	3.938	–	4.038	3.838
S.E. $(\widehat{\tau}_{\mathrm{F}})$	–	0.809	0.770	–	0.998	0.979	–	0.896	0.874
p-value	–	< 0.001	< 0.001	–	< 0.001	< 0.001	–	< 0.001	< 0.001

Table B3: Sensitivity analyses for NHEFS data with propensity scores determined by the probit model: a quadratic extrapolation function is used. The top panel reports the results of variable selection for the treatment model; the middle panel displays the estimation results of the ATE τ_0; and the bottom panel shows the estimation results of the ATE τ_0 by forcefully including "`age`" and "`sex`" to the selected variables to form the final treatment model to estimate τ_0.

Covariate	$R = 0.65$			$R = 0.75$			$R = 0.85$		
	full	LASSO	SCAD	full	LASSO	SCAD	full	LASSO	SCAD
intercept	-1.350	-1.863	-1.243	-1.173	-1.643	-1.062	-1.442	-1.909	-1.341
sbp	0.032	–	–	0.189	–	–	0.170	–	–
dbp	1.635	1.361	1.742	1.877	1.570	1.988	1.741	1.410	1.842
cholesterol	0.049	–	–	-0.057	–	–	0.078	–	–
price82	-0.790	-0.303	-0.303	-0.923	-0.393	-0.393	-0.744	-0.211	-0.211
ht	0.006	–	–	0.006	–	–	0.005	–	–
age	0.013	–	–	0.013	–	–	0.013	–	–
sex	0.032	–	–	0.034	–	–	0.023	–	–
nerves	-0.112	–	–	-0.129	–	–	-0.149	–	–
hbpmed	0.003	–	–	0.003	–	–	0.013	–	–
race	-0.597	-0.110	-0.110	-0.646	-0.116	-0.116	-0.624	-0.091	-0.091
$\widehat{\tau}$	2.500	3.457	3.215	1.944	3.335	3.008	2.149	3.586	3.319
S.E. ($\widehat{\tau}$)	1.503	1.312	1.259	1.399	1.074	0.975	1.224	0.942	0.923
p-value	0.318	0.006	0.014	0.499	< 0.001	< 0.001	0.348	< 0.001	< 0.001
$\widehat{\tau}_{\mathrm{F}}$	–	3.939	3.907	–	3.649	3.625	–	4.744	4.677
S.E. ($\widehat{\tau}_{\mathrm{F}}$)	–	1.076	0.904	–	1.050	0.888	–	0.826	0.800
p-value	–	< 0.001	< 0.001	–	< 0.001	< 0.001	–	< 0.001	< 0.001

Table B4: Sensitivity analyses for NHEFS data with propensity scores determined by the probit model: a linear extrapolation function is used. The top panel reports the results of variable selection for the treatment model; the middle panel displays the estimation results of the ATE τ_0; and the bottom panel shows the estimation results of the ATE τ_0 by forcefully including "`age`" and "`sex`" to the selected variables to form the final treatment model to estimate τ_0.

Covariate	$R = 0.65$			$R = 0.75$			$R = 0.85$		
	full	LASSO	SCAD	full	LASSO	SCAD	full	LASSO	SCAD
intercept	-1.752	-1.473	-1.702	-1.634	-1.343	-1.601	-1.942	-1.665	-1.907
sbp	0.078	–	–	0.111	–	–	0.134	–	–
dbp	0.639	0.488	0.594	0.550	0.382	0.446	0.643	0.473	0.560
cholesterol	-0.022	–	–	-0.005	–	–	-0.034	–	–
price82	-0.325	-0.046	-0.060	-0.288	-0.056	-0.045	-0.364	-0.087	-0.105
ht	0.006	–	–	0.007	–	–	0.005	–	–
age	0.013	–	–	0.013	–	–	0.012	–	–
sex	0.038	–	–	0.013	–	–	0.012	–	–
nerves	-0.092	–	–	-0.098	–	–	-0.104	–	–
hbpmed	0.004	–	–	0.006	–	–	0.004	–	–
race	-0.505	-0.226	-0.241	-0.530	-0.238	-0.315	-0.527	-0.250	-0.279
$\widehat{\tau}$	2.154	4.230	4.159	2.817	4.284	4.269	2.611	4.041	3.995
S.E. $(\widehat{\tau})$	1.115	1.075	1.062	1.098	0.872	0.863	1.289	1.253	1.234
p-value	0.301	< 0.001	< 0.001	0.098	< 0.001	< 0.001	0.211	0.001	0.001
$\widehat{\tau}_{\mathrm{F}}$	–	4.262	4.165	–	4.419	4.365	–	4.295	4.160
S.E. $(\widehat{\tau}_{\mathrm{F}})$	–	1.178	1.149	–	1.150	1.151	–	1.271	1.279
p-value	–	0.000	0.000	–	0.000	0.000	–	0.001	0.001

Table B5: Sensitivity analyses for NHEFS data with propensity scores determined by the probit model: a rational linear extrapolation function is used. The top panel reports the results of variable selection for the treatment model; the middle panel displays the estimation results of the ATE τ_0; and the bottom panel shows the estimation results of the ATE τ_0 by forcefully including "`age`" and "`sex`" to the selected variables to form the final treatment model to estimate τ_0.

Covariate	$R = 0.65$			$R = 0.75$			$R = 0.85$		
	full	LASSO	SCAD	full	LASSO	SCAD	full	LASSO	SCAD
intercept	-0.696	-0.660	-0.696	-0.708	-0.676	-0.711	-0.705	-0.667	-0.699
sbp	0.033	–	–	0.032	–	–	0.020	–	–
dbp	0.889	0.652	0.688	0.831	0.698	0.626	0.830	0.604	0.636
cholesterol	-0.008	–	–	-0.007	–	–	-0.009	–	–
price82	-1.069	-0.733	-0.733	-1.082	-0.650	-0.662	-1.048	-0.609	-0.609
ht	0.050	–	–	0.059	–	–	0.052	–	–
age	0.159	–	–	0.149	–	–	0.160	–	–
sex	0.006	–	–	0.004	–	–	0.007	–	–
nerves	-0.036	–	–	-0.038	–	–	-0.031	–	–
hbpmed	0.006	–	–	0.011	–	–	0.003	–	–
race	-0.785	-0.549	-0.585	-0.793	-0.461	-0.497	-0.781	-0.442	-0.475
$\widehat{\tau}$	1.057	3.854	3.612	1.364	3.388	3.748	1.546	3.677	3.280
S.E. $(\widehat{\tau})$	1.082	1.060	1.049	1.077	1.059	1.027	1.235	0.919	0.903
p-value	0.231	< 0.001	< 0.001	0.197	< 0.001	< 0.001	0.086	< 0.001	< 0.001
$\widehat{\tau}_{\mathrm{F}}$	–	3.867	3.763	–	3.568	3.442	–	4.071	3.390
S.E. $(\widehat{\tau}_{\mathrm{F}})$	–	0.891	0.885	–	1.163	1.146	–	0.992	0.957
p-value	–	< 0.001	< 0.001	–	0.002	0.003	–	< 0.001	< 0.001

Table B6: Sensitivity analyses for NHEFS data with propensity scores determined by the complement log-log model: a quadratic extrapolation function is used. The top panel reports the results of variable selection for the treatment model; the middle panel displays the estimation results of the ATE τ_0; and the bottom panel shows the estimation results of the ATE τ_0 by forcefully including "`age`" and "`sex`" to the selected variables to form the final treatment model to estimate τ_0.

Covariate	$R = 0.65$			$R = 0.75$			$R = 0.85$		
	full	LASSO	SCAD	full	LASSO	SCAD	full	LASSO	SCAD
intercept	-1.000	-1.024	-1.828	-1.485	-1.499	-1.372	-1.196	-1.092	-1.043
sbp	0.189	–	–	0.476	–	–	0.216	–	–
dbp	1.104	1.472	1.276	1.474	1.822	1.102	1.689	1.968	1.842
cholesterol	-0.099	–	–	-0.021	–	–	-0.134	–	–
price82	-0.885	-0.612	-0.603	-0.888	-0.589	-0.575	-1.094	-0.378	-0.245
ht	0.010	–	–	0.008	–	–	0.010	–	–
age	0.020	–	–	0.018	–	–	0.020	–	–
sex	0.097	–	–	0.048	–	–	0.081	–	–
nerves	-0.137	–	–	-0.184	–	–	-0.154	–	–
hbpmed	0.014	–	–	0.017	–	–	0.011	–	–
race	-0.961	-0.813	-0.675	-0.973	-0.749	-0.665	-0.952	-0.611	-0.503
$\widehat{\tau}$	1.989	3.566	3.675	1.848	3.986	3.861	2.169	3.700	3.173
S.E. $(\widehat{\tau})$	1.640	1.498	1.232	1.427	1.232	1.026	1.494	1.062	0.988
p-value	0.546	0.017	0.003	0.409	0.001	0.006	0.434	0.001	0.003
$\widehat{\tau}_{\mathrm{F}}$	–	4.300	4.881	–	4.257	3.955	–	3.898	3.815
S.E. $(\widehat{\tau}_{\mathrm{F}})$	–	1.081	0.955	–	1.276	1.200	–	1.095	0.908
p-value	–	< 0.001	< 0.001	–	0.002	< 0.001	–	0.005	0.001

Table B7: Sensitivity analyses for NHEFS data with propensity scores determined by the complement log-log model: a linear extrapolation function is used. The top panel reports the results of variable selection for the treatment model; the middle panel displays the estimation results of the ATE τ_0; and the bottom panel shows the estimation results of the ATE τ_0 by forcefully including "`age`" and "`sex`" to the selected variables to form the final treatment model to estimate τ_0.

Covariate	$R = 0.65$			$R = 0.75$			$R = 0.85$		
	full	LASSO	SCAD	full	LASSO	SCAD	full	LASSO	SCAD
intercept	-1.445	-1.013	-1.369	-1.593	-1.199	-1.523	-1.541	-1.060	-1.473
sbp	0.058	–	–	0.115	–	–	0.097	–	–
dbp	1.045	0.801	0.993	0.892	0.690	0.836	1.130	0.834	1.004
cholesterol	-0.006	–	–	-0.016	–	–	-0.062	–	–
price82	-0.385	-0.298	-0.334	-0.529	-0.136	-0.162	-0.391	-0.256	-0.279
ht	0.007	–	–	0.007	–	–	0.009	–	–
age	0.020	–	–	0.018	–	–	0.021	–	–
sex	0.051	–	–	0.066	–	–	0.039	–	–
nerves	-0.082	–	–	-0.174	–	–	-0.116	–	–
hbpmed	0.009	–	–	0.007	–	–	0.008	–	–
race	-0.811	-0.379	-0.409	-0.802	-0.409	-0.489	-0.769	-0.288	-0.313
$\widehat{\tau}$	0.884	3.798	3.691	0.869	3.906	3.809	1.558	4.087	3.974
S.E. $(\widehat{\tau})$	1.335	1.045	1.043	1.339	0.928	0.900	1.227	1.055	1.044
p-value	0.508	< 0.001	< 0.001	0.516	< 0.001	< 0.001	0.204	< 0.001	< 0.001
$\widehat{\tau}_{\mathrm{F}}$	–	3.861	3.695	–	4.005	3.973	–	4.110	4.055
S.E. $(\widehat{\tau}_{\mathrm{F}})$	–	1.160	1.207	–	1.190	1.206	–	1.092	1.114
p-value	–	0.002	0.001	–	0.001	0.001	–	0.000	0.000

Table B8: Sensitivity analyses for NHEFS data with propensity scores determined by the complement log-log model: a rational linear extrapolation function is used. The top panel reports the results of variable selection for the treatment model; the middle panel displays the estimation results of the ATE τ_0; and the bottom panel shows the estimation results of the ATE τ_0 by forcefully including "`age`" and "`sex`" to the selected variables to form the final treatment model to estimate τ_0.

Covariate	$R = 0.65$			$R = 0.75$			$R = 0.85$		
	full	LASSO	SCAD	full	LASSO	SCAD	full	LASSO	SCAD
intercept	-1.305	-1.251	-1.314	-1.292	-1.218	-1.306	-1.311	-1.246	-1.331
sbp	0.065	–	–	0.061	–	–	0.057	–	–
dbp	0.271	0.204	0.262	0.210	0.123	0.173	0.161	0.086	0.090
cholesterol	-0.010	–	–	-0.016	–	–	-0.011	–	–
price82	-0.068	-0.013	-0.016	-0.065	-0.029	-0.019	-0.063	-0.025	-0.022
ht	0.092	–	–	0.084	–	–	0.072	–	–
age	0.232	–	–	0.232	–	–	0.217	–	–
sex	0.010	–	–	0.003	–	–	0.014	–	–
nerves	-0.048	–	–	-0.055	–	–	-0.039	–	–
hbpmed	0.015	–	–	0.004	–	–	0.011	–	–
race	-0.279	-0.225	-0.288	-0.293	-0.219	-0.307	-0.273	-0.208	-0.292
$\widehat{\tau}$	0.628	2.656	2.905	1.575	3.770	3.808	2.003	3.544	3.386
S.E. $(\widehat{\tau})$	1.266	1.101	1.020	1.286	1.051	1.026	1.886	1.442	1.390
p-value	0.620	0.009	0.008	0.221	< 0.001	< 0.001	0.288	0.011	0.019
$\widehat{\tau}_{\mathrm{F}}$	–	3.437	3.294	–	3.850	3.694	–	3.627	3.484
S.E. $(\widehat{\tau}_{\mathrm{F}})$	–	1.162	1.169	–	0.982	0.956	–	0.885	0.898
p-value	–	0.003	0.004	–	< 0.001	< 0.001	–	< 0.001	< 0.001
